# Supplementary material for: MRP3 is a sex determining gene in the diatom Pseudo-nitzschia multistriata
Source: Nat Commun. 2018 Nov 28;9:5050. doi: 10.1038/s41467-018-07496-0 (PMC6261938; doi:10.1038/s41467-018-07496-0)
Supplement: Supplementary file 1 — Supplementary Information [file 41467_2018_7496_MOESM1_ESM.pdf]

Supplementary Information

MRP3 is a sex determining gene in the diatom *Pseudo-nitzschia multistriata*

Russo et al.

Supplementary Table 1. List of genes differentially expressed between MT+ and MT- samples in the RNA-seq study. In bold the candidate genes tested in qPCR; the five mating type related genes are underlined and marked with (^). The gene ID, logFC (logarithmic Fold Change) in RNA-seq and qPCR, p-Value, FDR (False Discovery Rate), Description of the predicted protein and the CPM (counts per million) for each RNA-seq library are reported. For the RNA-seq libraries (last nine columns), the strain name is reported above the library code (see also Supplementary Table 5). The table continues on the following page.

						MT+					MT-			
Gene ID	logFC	qPCR FC ± variance	p-Value	FDR	Description	B938 Lib41	B856 Lib74	B856 Lib HCUH	Sy373 Lib CIIO1	Sy373 Lib CIIO2	B857 Lib45	B857 Lib HCUN	B939 Lib77	Sy379 Lib CIIP2
0122210	8.65		2.86E-06	3.28E-03	-	0.00	0.81	2.78	1.79	0.90	0.00	0.00	0.00	0.00
**0020770^**	**8.53**	**7.5 ± 5**	**8.46E-22**	**1.00E-17**	**-**	**7.80**	**14.22**	**8.68**	**10.32**	**16.16**	**0.09**	**0.00**	**0.04**	**0.00**
0109190	7.22		6.64E-14	2.63E-10	-	2.30	1.47	2.36	1.98	3.08	0.02	0.00	0.03	0.00
0060210	7.11		3.55E-05	1.76E-02	-	0.00	0.10	0.22	0.40	0.61	0.00	0.00	0.00	0.00
0022790	6.49		1.15E-05	6.86E-03	116 kDa U5 small nuclear ribonucleoprotein component	0.12	0.04	0.28	1.11	0.51	0.00	0.01	0.00	0.00
0046260	6.27		1.04E-05	6.47E-03	-	1.09	0.67	0.04	5.98	2.21	0.05	0.01	0.00	0.05
0104260	6.11		1.04E-04	4E-02	-	0.03	3.65	6.95	0.76	0.87	0.00	0.06	0.00	0.08
0042070	5.36		1.98E-06	2.61E-03	-	3.76	1.70	2.69	4.87	3.93	0.00	0.32	0.00	0.00
0106620	4.93		2.12E-05	1.20E-02	-	5.45	1.62	0.16	4.82	7.85	0.18	0.01	0.27	0.06
0060410	4.57		2.00E-07	3.40E-04	Phosphatidylinositol 4-phosphate 5-kinase 9	0.56	0.63	0.30	1.36	1.16	0.03	0.02	0.04	0.05
0036200	4.40		3.95E-06	3.71E-03	Delta(12) fatty acid desaturase fat-2	8.64	3.56	3.13	41.55	58.31	1.91	0.77	1.20	0.59
**0122240^**	**4.33**	**3.7 ± 2.6**	**4.88E-13**	**1.45E-09**	**LRR receptor-like serine/threonine-protein kinase GSO1**	**54.12**	**166.19**	**105.89**	**113.59**	**143.65**	**4.97**	**11.03**	**4.83**	**3.82**
0108790	3.84		4.33E-06	3.71E-03	-	6.91	3.05	1.57	5.84	2.35	0.20	0.39	0.47	0.07
**0024820^**	**3.06**	**9.8 ± 3**	**1.10E-04**	**4.06E-02**	**-**	**173.97**	**306.15**	**214.05**	**181.47**	**225.29**	**25.26**	**4.72**	**65.51**	**10.19**
0068000	2.40		7.79E-06	5.61E-03	Elongation of very long chain fatty acids protein 5	19.39	25.93	19.36	22.20	28.41	3.45	7.52	5.69	1.59
0113990	2.39		9.98E-05	4E-02	-	11.87	5.32	12.44	4.00	6.78	1.21	2.68	1.84	0.65
0025090	2.07		4.88E-05	2.23E-02	-	17.00	26.45	20.64	39.55	27.55	8.35	9.32	6.86	2.17
0091290	1.91		3.39E-05	1.75E-02	-	31.09	21.97	24.83	27.49	42.03	13.60	7.36	9.16	3.41

						MT+					MT-			
Gene ID	logFC	qPCR FC ± variance	p-Value	FDR	Description	B938 Lib41	B856 Lib74	B856 Lib HCUH	Sy373 Lib CIIO1	Sy373 Lib CIIO2	B857 Lib45	B857 Lib HCUN	B939 Lib77	Sy379 Lib CIIP2
**0085380^/ 0041130**	*	**-6.6 ± 0.8**			**Heat shock factor protein 3**	0	0	0	0	0	0	0	0	0
**0006960^**	**-8.17**	**-9.7 ± 4.6**	**1.76E-16**	**1.04E-12**	**Probable leucine-rich repeat receptor-like protein kinase At1g35710**	**0.58**	**1.21**	**0.20**	**0.09**	**0.18**	**101.66**	**179.60**	**232.77**	**59.40**
0081520	-8.04		4.44E-10	8.79E-07	Myb-related protein Zm38	0.00	0.00	0.00	0.00	0.00	2.22	0.65	0.40	1.08
0052370	-6.21		1.04E-04	4E-02	Phosphoenolpyruvate carboxykinase ATP	0.46	1.06	1.11	3.50	6.95	4.34	0.80	8.63	689.14
0020420	-5.11		8.45E-07	1.25E-03	-	3.18	2.86	0.68	0.74	1.13	4.33	186.77	56.10	10.26
0103000	-4.72		7.36E-06	5.61E-03	Cathepsin D	1.37	1.75	0.06	0.97	1.50	4.76	63.40	52.07	2.92
**0045400**	**-4.60**	**0.2 ± 1.4**	**1.01E-05**	**6.47E-03**	**Putative oxidoreductase YteT**	**2.04**	**0.74**	**6.14**	**5.04**	**8.54**	**31.63**	**17.37**	**13.04**	**342.54**
0045410	-4.57		6.59E-05	2.90E-02	Probable NAD(P)H-dependent D-xylose reductase xyl1	1.23	0.39	5.18	6.90	8.90	30.75	9.37	13.73	342.07
**0021670**	**-4.20**	**0 ± 1.4**	**3.04E-06**	**3.28E-03**	**-**	**3.18**	**2.06**	**2.26**	**3.48**	**5.26**	**29.99**	**3.23**	**23.23**	**174.72**
**0093550**	**-4.01**	**0 ± 1**	**8.04E-06**	**5.61E-03**	**-**	**36.61**	**1.00**	**5.36**	**11.26**	**12.39**	**223.97**	**39.82**	**279.09**	**320.40**
**0084970**	**-3.05**	**0 ± 43**	**1.84E-10**	**4.37E-07**	**-**	**1.64**	**1.05**	**2.52**	**2.61**	**1.71**	**11.81**	**21.78**	**10.58**	**20.34**
0076430	-2.96		1.41E-04	4.82E-02	Glutamate synthase large subunit-like protein YerD	14.83	1.24	6.34	8.94	13.21	53.01	9.63	75.93	130.69
0057230	-2.81		4.66E-05	2.21E-02	-	0.92	1.60	1.45	3.33	2.06	25.46	8.90	2.83	20.12
0004160	-2.51		3.06E-05	1.65E-02	-	7.24	3.43	8.79	4.84	7.38	19.65	9.73	44.16	65.34
0038950	-2.41		1.30E-04	4.69E-02	-	4.40	1.67	12.32	2.32	3.49	25.58	21.24	43.10	12.16
0072100	-2.27		1.42E-04	4.82E-02	Tetracycline resistance protein class A	6.30	3.41	2.75	7.18	5.25	26.61	5.47	43.26	21.20
0078000	-2.19		7.61E-05	3.23E-02	-	1.95	1.99	5.21	1.47	2.20	10.47	10.75	18.06	7.90
0113630	-2.03		4.37E-06	3.71E-03	Protein cereblon	2.76	1.64	2.32	2.92	2.08	15.44	12.96	6.17	7.22

* This gene (two identical gene models) was not present in the output of the differential expression analysis because of a filter on multimapping reads. It has been tested based on its expression profile during the early stages of *P. multistriata* sexual reproduction[1].

Supplementary Table 2. *P. multistriata* strains used in this study. The strain name, mating type defined in experimental crosses, isolation date, origin (environment or lab crosses) and the bands obtained in the genotyping PCR are given, along with the information on whether a given strain has been used for the qPCR analyses, for RNA-seq and/or to clone and sequence the alleles. >SST or < SST = above or below the sexualization size threshold.

Strain name	Mating Type	Isolation date	Origin/Parental strains	Genotyping PCR result	qPCR	RNA-seq	Alleles cloned and sequenced
B935	+	24/05/2012	isolated from water sample		X		
SH18	+	07/04/2013	Sy776-*SP2+		X		
MVR1041.6	+	05/02/2013	isolated from water sample		X		
MVR171.8	+	07/06/2013	isolated from water sample		X		
B936	-	24/05/2012	isolated from water sample	MB	X		
SH20	-	07/04/2013	Sy776-*SP2+		X		
MVR1041.4	-	05/02/2013	isolated from water sample	M	X		
MVR171.1	-	07/06/2013	isolated from water sample		X		
1119-15	+	02/09/2014	isolated from water sample	AM			X
1120-32	-	09/09/2014	isolated from water sample	M			X
1078-30	+	28/10/2013	isolated from water sample	AB			X
1120-47	-	02/09/2014	isolated from water sample	MB			
1120-48	-	09/09/2014	isolated from water sample	MB			X
1075-25	+	08/10/2013	isolated from water sample	AB			
1120-5	+	02/09/2014	isolated from water sample	A			
1120-25	+	02/09/2014	isolated from water sample	AB			
1120-7	-	02/09/2014	isolated from water sample	B			
1120 14	-	02/09/2014	isolated from water sample	B			
Sy373	+	07/072009	isolated from water sample	AM		X	
Sy379	-	07/07/2009	isolated from water sample			X	
B854 (A13)	-	13/10/2010	Sy373xSy379	M			
B855 (A1.6)	+	13/10/2010	Sy373xSy379	AB			
VF2.2.9/11	-	02/08/2011	B855 x B854	MB			
B857 (VF2.5.11)	-	02/08/2011	B855 x B854	MB		X	X
VF2.1.9	+	02/08/2011	B855 x B854	AM			
B856 (VF2.3.5)	+	02/08/2011	B855 x B854	AM		X	X
PmF3.2	+	2012	B856 x ?	A			
LV 77	-	2015	B855 x MVR1041.4	B			
LV 80	+	2015	B855 x MVR1041.4	AM			
LV 84	-	2015	B855 x MVR1041.4	B			
LV 88		2015	B855 x MVR1041.4	B			
LV 89	+	2015	B855 x MVR1041.4	A			
LV 91	+	2015	B855 x MVR1041.4	AM			
LV 92A	-	2015	B855 x MVR1041.4	MB			

Supplementary Table 2 continued

LV 106		2015	B855 x MVR1041.4	B			
LV 112		2015	B855 x MVR1041.4	B			
LV 113	-	2015	B855 x MVR1041.4	B			X
LV 117	-	2015	B855 x MVR1041.4	MB			
LV 121	+	2015	B855 x MVR1041.4	AM			X
LV 122	-	2015	B855 x MVR1041.4	MB			
LV 123	-	2015	B855 x MVR1041.4	B			
LV 125	+	2015	B855 x MVR1041.4	AM			
LV 127	+	2015	B855 x MVR1041.4	AM			
LV 129	-	2015	B855 x MVR1041.4	B			
LV 130	+	2015	B855 x MVR1041.4	A			X
LV 136	-	2015	B855 x MVR1041.4	B	X	X	
LV 141	+	2015	B855 x MVR1041.4	A			
LV 142	-	2015	B855 x MVR1041.4	MB			
LV 148	-	2015	B855 x MVR1041.4	B			
LV 149	+	2015	B855 x MVR1041.4	AM			
LV 162	+	2015	B855 x MVR1041.4	AM			
LV 164	-	2015	B855 x MVR1041.4	MB			
LV 168	+	2015	B855 x MVR1041.4	A			
LV 169	-	2015	B855 x MVR1041.4	MB			
LV 175	+	2015	B855 x MVR1041.4	A			
LV 177	+	2015	B855 x MVR1041.4	AM			X
LV 179	+	2015	B855 x MVR1041.4	A			
LV 180	+	2015	B855 x MVR1041.4	A			
LV 181	-	2015	B855 x MVR1041.4	B			
LV 182	+	2015	B855 x MVR1041.4	A			
LV 186	-	2015	B855 x MVR1041.4	MB			
LV 193	-	2015	B855 x MVR1041.4	B			
LV 195	+	2015	B855 x MVR1041.4	AM			
MC 1217 (11)	+	13/09/2016	isolated from water sample	-			
MC 1217 (13)	-	13/09/2016	isolated from water sample	-			
MC 1217 (17)	-	13/09/2016	isolated from water sample	M/B			
F4 B1 >SST	NC. - when <SST	06/12/2016	PmF3.2 x MC 1217 13	M			
F4 B2 >SST	NC. - when <SST	06/12/2016	PmF3.2 x MC 1217 13	M			
F4 B3 >SST	NC. + when <SST	06/12/2016	PmF3.2 x MC 1217 13	AM			
F4 B4 >SST	NC	06/12/2016	PmF3.2 x MC 1217 13	AM			

Supplementary Table 2 continued

F4 B5 >SST	NC	06/12/2016	PmF3.2 x MC 1217 13	M			
B6 >SST	NC	06/12/2016	MC 1217 11 x MC 1217 13	AM			
B7 >SST	NC	06/12/2016	MC 1217 11 x MC 1217 13	MB			
B8 >SST	NC	06/12/2016	MC 1217 11 x MC 1217 13	AM			
B9 >SST	NC. - when <SST	06/12/2016	MC 1217 11 x MC 1217 13	MB			
B10 >SST	NC. + when <SST	06/12/2016	MC 1217 11 x MC 1217 13	AM			
Sy682	-	07/09/2010	isolated from water sample	MB			
Sy799	-	21/09/2010	isolated from water sample	B			
Sy800	+	21/09/2010	isolated from water sample	B			
Sy798	+	21/09/2010	isolated from water sample	AM			
MM1A1	NT	2011	isolated from water sample	M			
ES814	+	2011	isolated from water sample	AB			
1068 13	-	20/08/2013	isolated from water sample	B			
1068 14	-	20/08/2013	isolated from water sample	B			
1068 33	+	20/08/2013	isolated from water sample	AB			
1068 81	-	20/08/2013	isolated from water sample	MB			
1070 14	+	03/09/2013	isolated from water sample	AB			
1070 13	+	03/09/2013	isolated from water sample	AB			
1068 57	+	20/08/2013	isolated from water sample	AB			
1264 B3a	+	29/08/2017	isolated from water sample	AM			
1264 B4	+	29/08/2017	isolated from water sample	AB			
1266 B3a	-	13/09/2017	isolated from water sample	B			
1266 B4b	+	13/09/2017	isolated from water sample	AB			
KA105 >SST	NC	28/06/2017	MC1217-17 x MC1217-5	AM			
KA204 >SST	NC	28/06/2017	MC1217-17 x LV168	AB			
MR3 >SST	NC	21/06/2017	LV92A5 x PMF3.2	MB			
MR5 >SST	NC	21/06/2017	LV92A5 x PMF3.3	AB			

NC= non-competent for sex because cell size was above sexualization threshold.
NT = not tested, dead, only gDNA available.

Supplementary Table 3. Cycle Threshold (CT) values (technical triplicates and average) from qPCR amplification of the control gene tubulin-β (TUB B) and of the gene 0020760.

Strain name and MT	TUB B	TUB B average CT	20760	20760 average CT
**SP4 MT-**	21.43401	21.397765	27.72913	27.575966
	21.3896		27.54986	
	21.36968		27.4489	
**SH20 MT-**	22.10721	22.367543	29.3734	29.55899
	22.29175		30.14509	
	22.70367		29.15848	
**41.4 MT-**	19.07514	19.224321	24.45341	24.456882
	19.3061		24.44812	
	19.29172		24.46911	
**171.1 MT-**	21.69194	21.717037	33.27479	33.123839
	21.77249		33.12741	
	21.68668		32.96932	
**SP2 MT+**	22.96304	22.933743	36.44473	37.115078
	22.96545		37.78543	
	22.87274		ND	
**SH18 MT+**	21.73822	21.712673	27.16025	27.188158
	21.91142		27.05754	
	21.48838		27.34668	
**41.6 MT+**	20.25243	20.28755	32.44352	32.619483
	20.33863		32.15172	
	20.27159		33.26321	
**171.8 MT+**	23.14835	23.120984	29.08989	29.242554
	23.03572		29.07984	
	23.17888		29.55793	

ND, not determined

Supplementary Table 4. List of genes differentially expressed in the transgenic strain LV136T3 compared to the wild type strain LV136. Two new putative MR genes are indicated in bold.

Gene ID	logFC	logCPM	PValue	FDR	Gene name
0020770.1	10.84	4.77	5.763E-21	5.397E-17	*MRP3*
0024810.1	10.40	9.18	1.316E-11	3.081E-08	*MRP1*
0122240.1	4.57	6.65	1.161E-08	2.174E-05	*MRP2*
0070330.1	-2.62	5.17	2.313E-05	2.708E-02	***MRX1***
0058000.1 *	-3.27	3.53	3.516E-05	3.297E-02	***MRX2***
0036960.1 *	-3.27	3.53	3.520E-05	3.297E-02	***MRX2***
0041130.1 °	-5.88	3.72	3.889E-08	5.203E-05	*MRM1*
0085380.1 °	-5.88	3.72	3.868E-08	5.203E-05	*MRM1*
0006960.1 ^	-6.37	5.77	6.725E-13	2.099E-09	*MRM2*
0006960.2 ^	-6.37	5.77	6.705E-13	2.099E-09	*MRM2*

Gene models marked with identical symbols are either isoforms (^) or are identical but map on two different scaffolds (* and °).

Supplementary Table 5. Libraries selected for the differential expression analysis with corresponding strain code, strain Mating Type (MT), accession number, reference, sequencing strategy, number of reads before and after the quality check (QC).

**Library Name**	**Strain code**	**MT**	**Accession number**	**Reference**	**Strategy**	**Reads before QC**	**Reads after QC**
41	B938	+	E-MTAB-5469	[1]	Single-end	28.458.025	28.341.225
45	B857	-	E-MTAB-5469	[1]	Single-end	34.070.601	33.766.714
74	B856	+	E-MTAB-5469	[1]	Single-end	19.270.248	19.194.720
77	B939	-	E-MTAB-5469	[1]	Single-end	27.925.811	27.652.379
CIIO1	Sy373	+	SRX059292	Unpublished	Paired-end	42.736.393	35.243.577
CIIO2	Sy373	+	SRX115126	[2]	Paired-end	26.423.748	22.914.921
CIIP2	Sy379	-	SRX115125	[2]	Paired-end	30.856.367	20.823.225
HCUH	B856	+	SRX1070747	Unpublished	Paired-end	54.545.126	50.297.563
HCUN	B857	-	SRX1070749	Unpublished	Paired-end	52.851.954	48.670.573

Supplementary Table 6. List of primers used in this study and corresponding sequences.

Primer name	Location	Primer sequence 5'-3'
F147prom	*MRP*3 promoter	GGCAACAAGTGCTTGCTAATG
R147prom	*MRP*3 promoter	GATGATGTACAACAATCGGCG
R147p2	*MRP*3 promoter	GGGTCTACGTACGGTATGGTG
F147p2	*MRP*3 promoter	CAATCCTACATAGGCCCAATATC
SC432promFW	*MRM1* promoter	GAGTTCTCTTGCCGGATGATAC
SC432promRV	*MRM1* promoter	CCCTCATTCACCACATGTGAC
0020770+ F	*MRP*3 coding region	GCGCAAGCAATCTAAGGTG
0020770+ R	*MRP*3 coding region	GACGTCGACGGCTATTTTG
F147p3	*MRP*3 promoter	GGATCCTTTGAGCAACACAG
R147p3	*MRP*3 promoter	CCAAGTGATGCTGCATACAAG
0.00+F	*MRP1* coding region	GTATGGCGCTCACCACTTC
0.00+R	*MRP1* coding region	CGTCTTCGACTGCGTCTTC
127.15 F3	*MRP2* coding region	CCTCCGAATATGGATACATG
127.15 R3	*MRP2* coding region	GAGCTAAACATCGTGACACC
47507F	*MRM1* coding region	CCCCTACAAGCTCTTTGATTTG
47507R	*MRM1* coding region	GAAATTGTGGTGCCCAAAG
46228F	*MRM2* coding region	CCACCGAACTAGGCAACTGTC
46228R	*MRM2* coding region	GGCACAGAACCCGTCAAC
FMrp3Eco	*MRP*3 expressing vector	GGCGGCCGAATTCATGAATGACGAATCGAAT
RMrp3Sma	*MRP*3 expressing vector	GGCGGCCCCCGGGCTACATCTGCTGCATTAG
FPIH4	NAT expressing vector	AAAGCTACTAGTGGCCATTTTGGAATTTGTCG
RPIH4	NAT expressing vector	TCGACTACCTCAGTCTTCGGCTCTAGAATGACC
FPIIH4	NAT expressing vector	TCGGCTCTAGAATGACCACTCTTGACGACACG
RPIIH4	NAT expressing vector	TCGACTACCTCAGTCTTCGGCTCTAGAATGACC
H4up1	*MRP*3 and NAT expressing vectors	GTGGGAATGAACAGAACGAGA
NATfor	NAT expressing vector	TGACCACTCTTGACGACACG
NATrev	NAT expressing vector	GTTGACGTTGGTGACCTCC
tArev	*MRP*3 and NAT expressing vectors	CCCAGCCAAAGTCGAGGTAG

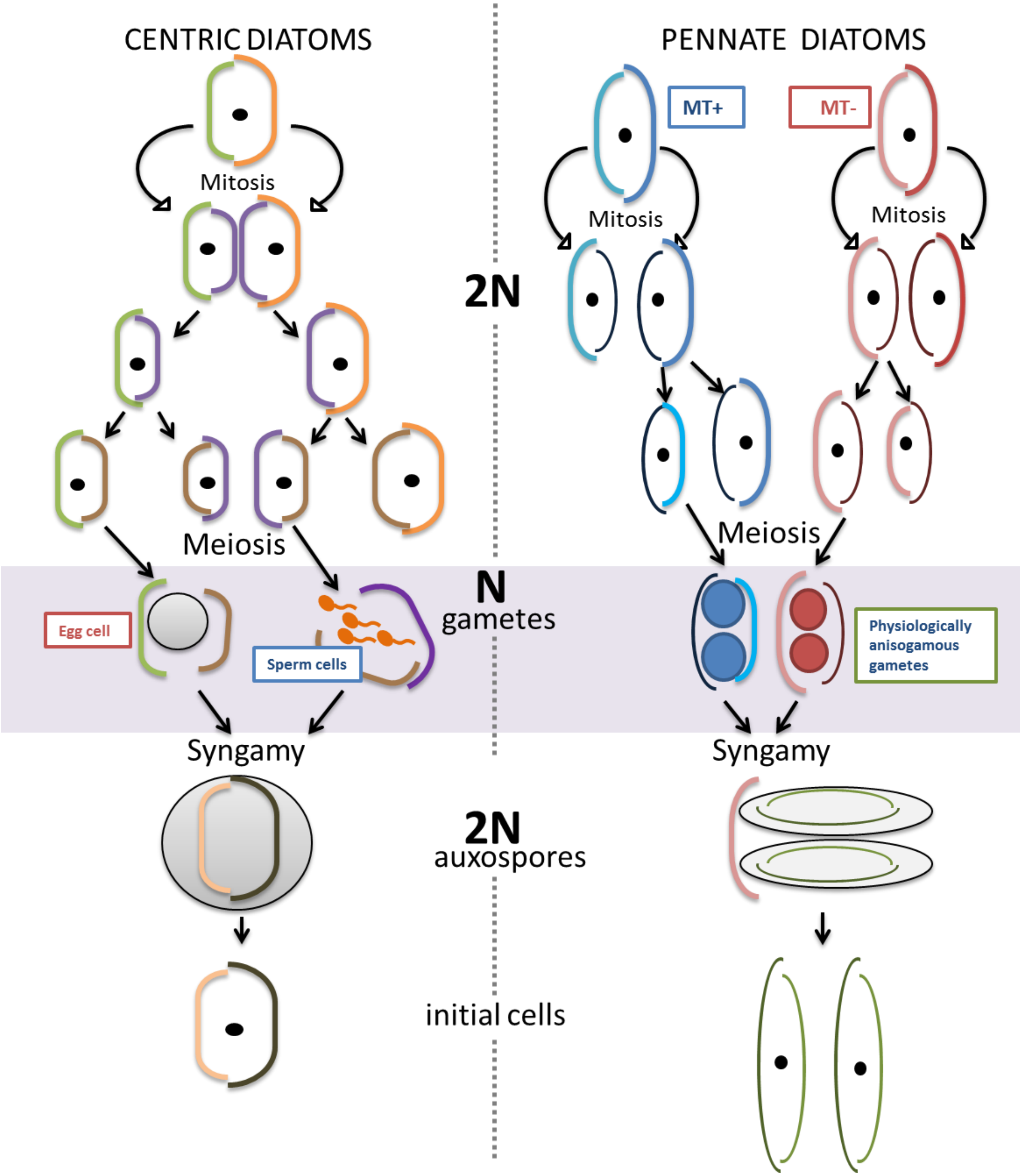

Supplementary Figure 1. Schematic representation of the life cycle in centric (left) and pennate (right) diatoms. Diatoms are diploid (2N) and gametes are the only haploid (N) stage in their life cycle. Centric diatoms are homothallic and anisogamous, while pennate diatoms are mostly heterothallic and isogamous or physiologically anisogamous. Auxospores, not surrounded by the rigid siliceous frustule, can expand, and within them the maximum sized initial cell is produced. This cell will start dividing mitotically.

SignalP output

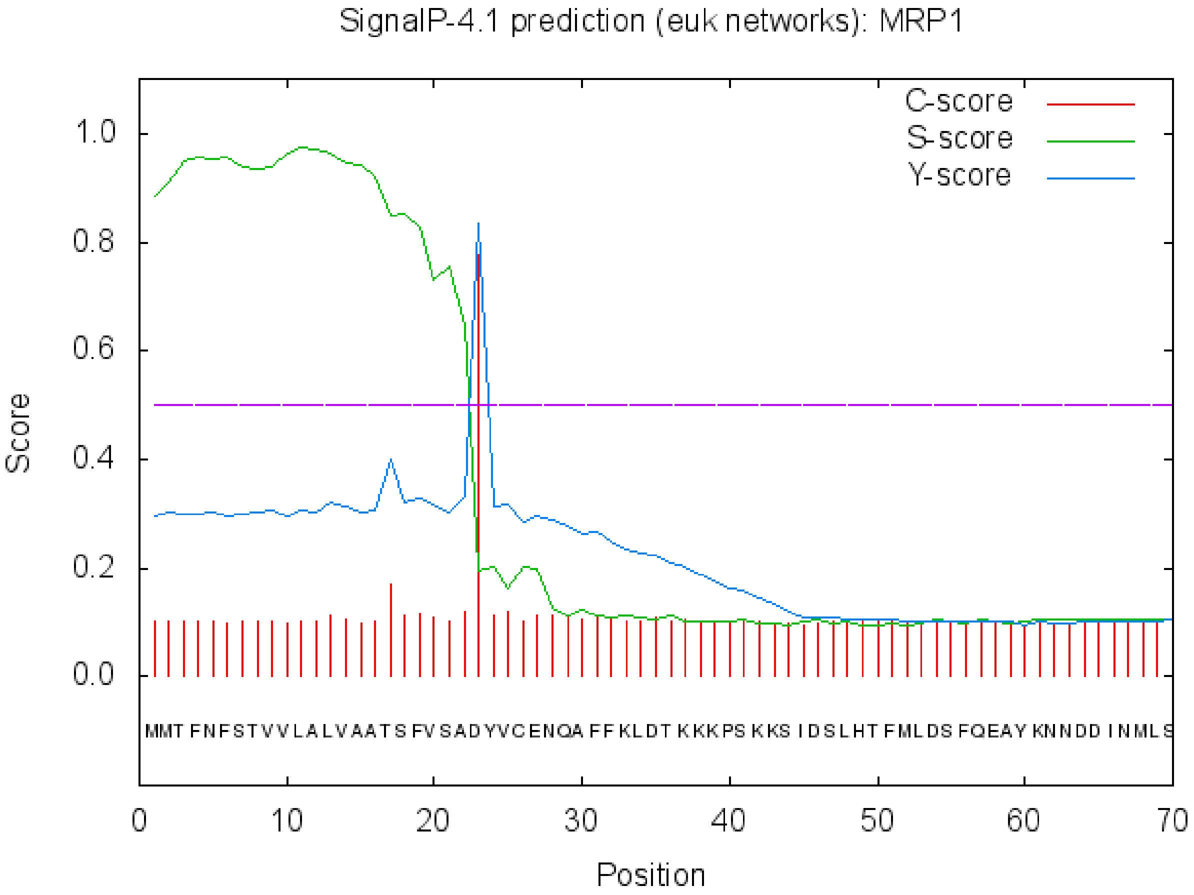


```
# Measure  Position  Value    Cutoff   signal peptide?
  max. C    23        0.778
  max. Y    23        0.836
  max. S    11        0.975
  mean S     1-22     0.899
       D     1-22     0.870     0.450   YES
Name=MRP1       SP='YES' Cleavage site between pos. 22 and 23: VSA-DY D=0.870
D-cutoff=0.450 Networks=SignalP-noTM
# data
# gnuplot script
```

AsaFind output

```
This is ASAFind version 1.1.5.
You used SignalP-4.1.
You submitted 1 proteins
0 of your proteins were SignalP negative
1 of your proteins were SignalP positive
    0 of these were predicted to go to the plastid
        0 of these were predicted with high confidence
        0 of these were predicted with with low confidence
```

Supplementary Figure 2. Outputs of prediction software for transit peptides for the MRP1 protein. Top, output of the SignalP 4.0 software, which can predict the presence of a secretory signal peptide, a ubiquitous protein sorting signal that targets proteins for translocation across the endoplasmic reticulum (ER) membrane. Bottom, output of the AsaFind software, a prediction tool that identifies proteins with a signal peptide for transport to diatom plastids. The MRP1 protein contains a signal peptide but it is not predicted to go to the plastid.

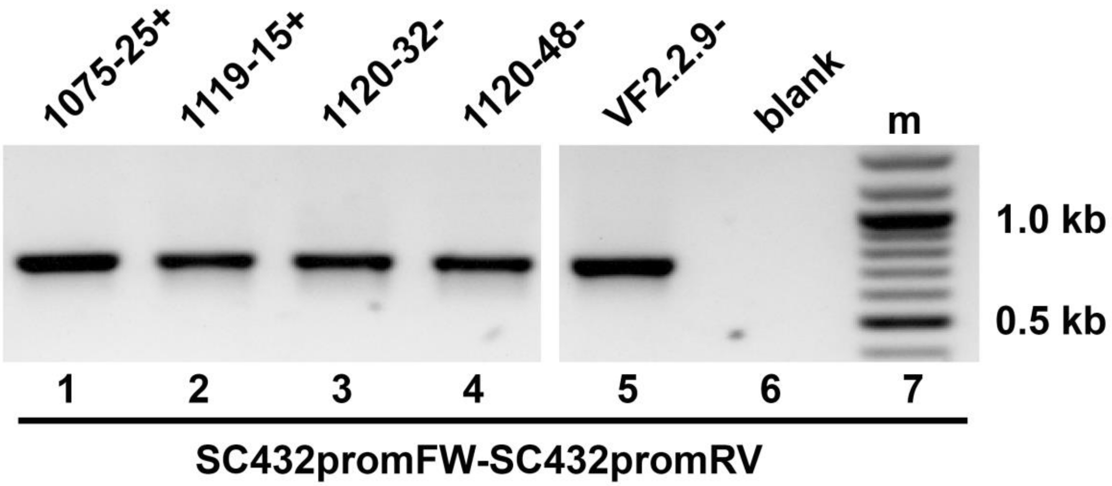

Supplementary Figure 3. PCR amplification of a 728 bp region upstream of the *MRM1* gene. Electrophoretic gel with amplicons obtained from two MT+ and three MT- strains.

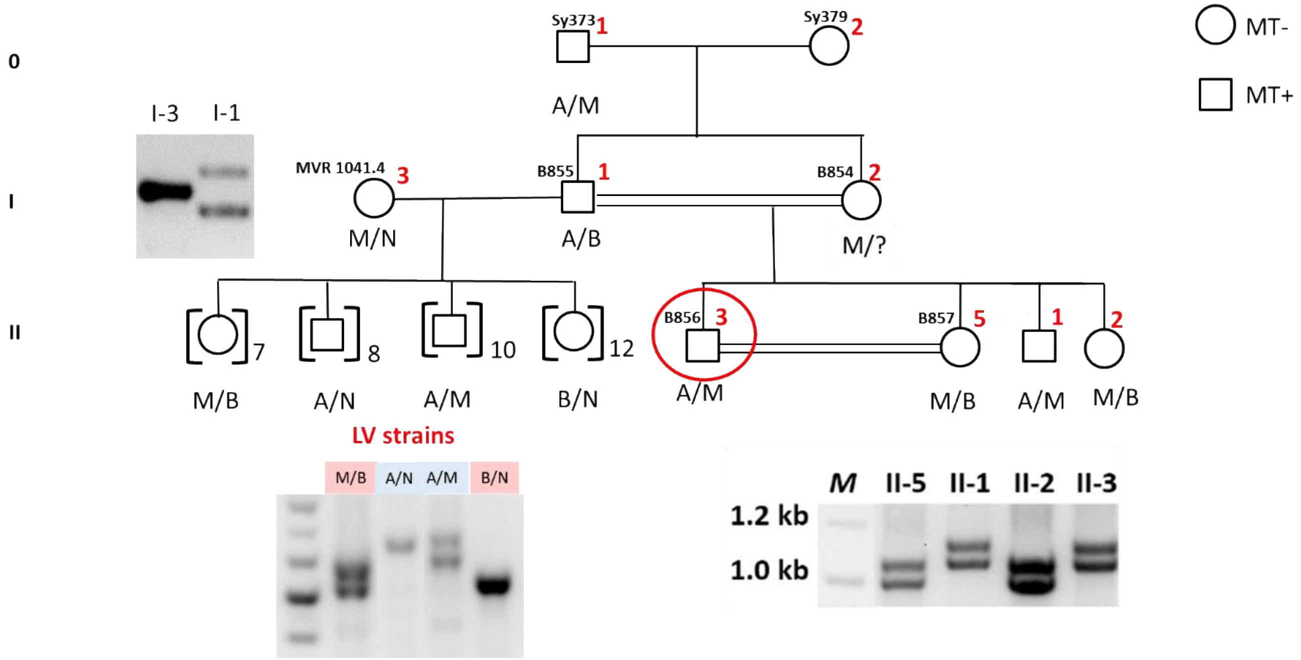

Supplementary Figure 4. The A allele segregates with the MT+ phenotype. *P. multistriata* pedigree with three generations. Each individual is represented by a square for MT+ or a circle for MT- and is defined by the generation number (in Roman on the left panel) and a progressive Arabic number (in red). Strain names are reported above each square or circle when available. The alleles of each individual are indicated below the symbol. The question mark indicates uncertainty on the second allele. In generation II subscript numbers next to brackets indicate how many individuals of that MT with that genotype were obtained from the cross of I-1 and I-3. Electropherograms show bands obtained from a selection of individuals in the pedigree. For all other genotypes refer to Supplementary Table 2. The strain circled in red was used to produce the reference genome[1].

```
>A_allele

AATAATTAGCTATACTACTACTACTACTACTACTACTACTACTACTACTACTACTAC
TACTACTACTACTACTACTACTACTACTACTACTACTACTACTACTACTACTACTAC
TACTACTACTACTACTACTACTACTACTACTACTACTACTACTACTACTACTACTAC
TACTAGGAGTACTAGTAGTAGTGGGCAGTACTACTAGTAGTAGCTACTAATAGTACTAAT
AGTACTATTAGTGGTGGTCTTACTACTACTAGTACTAGTAGCACCAGTCGTAGTACTAGC
AAAGTACAGCAGTACTACTACTAGTTATTACTAGTAGTACTAGTAGTACTAGACAAGTAA
TATAGCAGTACTAATAGTGGTAGTGAAATAAGTTTCTGCTATTCATTGTACAATCCTACA
TAGGCCCAATATCAATGACAAACATTTAGGAAAATCAAAATAATGAAGATACAATGACAC
ATACTATGGGAACAGAGGCAAGTACCACCTAAAGACACTTGTTATTATTGTACTCATAAC
CGATTCCAGATTCGTGCCACACGTTCCTATCTACCACCATACCGTACGTAGACCCACATC
AAGTAAACAATTAATGGGGTCAAGTAAGAAACTTGAAAACTACCATTACCAACACAACCA
GAGAAATGGCGTTTATTCTTGTTTTTGTTTCTGACGGCTTCTGCACAATGGTAGCAACGT
AGATTAAAAGAGGACTGAACCAATTGAATCGCTGATTGGACCCGGGAGCCTTTTTTGTTC
AAGACACTGGACAAGGCTTAAACGATTTTTGAGTAGGAGTATGTGCCTTTTTTCTTCGAT
CACAGATCATGTTCAAGGAGGAAAGAATAATACTACGAGTAATATTGTGAATAGAACCAC
GATAAAAACGAATACAACATCACAGCACAAGGCATGGCATTGTACTCAGCATAGCACAAC
ACACCGAACAACACATAAAAGCCATTTCACTACACTtgtaAGCTCCGAAATCAATCAGTA
CAATTCATCGCCGATTGTTGTACATCATCATTTGTCTACTCCATATTACAAACAAGTGAC
AAAAAGAAAAAAAGATCTTTAGTCAAAATGAATGACGAATCGAATAAAGAATGGACCTAC
TACTTCGGTCGAGGAGGCGCTTGCAACCAACAGCGCAAGCAATCTAAGGTGGTCGGTTTG
TTGCAGAAACTGGCTCTCCGCTATAACCAGTGCCATCGAGAAGAGAAAAGACTTTTTGCG
AAATGCGAAGTCTATAATATAGTTCTTGAAAAGGGAGGTTCGTTTTTCGAAATAACGAAC
AAAATAGCCGTCGACGTCACGGCGGACGAAATGAAATCAACTACAAAGATCATGCAAGTT
TTTCGAGATATAAATAAACGTAACAAGAAGAGTAAAGGGTCTGCAAAGGCGAAAAAGAAG
CCATCTTTCACTTCTCGAACGAAAAAAAATAAGCCGTCTTTTTGCACTTTACAAACTACT
AGAGCAAGAGAATCACGAACGACCAAAATGAAAATAAAAACTCAACCAAAACGCAGGTGC
GTCAAGCCTAGCATAATTGAAGCGCCAATCATGAATTGTGTGAGGGATGCTTTACTGATG
GCCAGCGCGAAAAGTCAATCGCTTCCTGACACAAGGACAAACAAGCCGGGGTTGGGTACT
CCGGTCAGGGGTAACGGATCCGAGCTGAAGCGACTTATTGCGGAAAGTAATCATCGCTTC
TCAACAAATAATGAAACATTTGATTTAATGGCCGTGGAACCACCTCAGCTAGAGAACTCG
TTTAGTGCACTGATCATGGCGGATGAGGTAACAACGGAACTTCCAACTGATTCTCAAATG
AGTGTGAACCTACACCTGCACAAAAGAGTACAGCGACTAGAAAATCTTGTTGGGATGCTA
ATGCAGCAGATGtagAGAGTCTAGTGTAGCTAAATACTTAAACATGGAAACAGGAATCAC
TCGACGCTACGTGTATCAAAAAATATGCAGCGATCGGATTTGCGCAACACAATCTGCTCT
CTGGGCTGGCATCAAATTCCAAGGAAAATGTTCCGCCCGATGGTGACTACTTTGACTAAA
AAACTTGCTTGAACTCCTGTTACAGCAAAATCAGATGCGCGTGAAAATATTTGGTCAGCA
AGAGCACGCAAAACTATTTGGTTGCAAGGAATAAAATTTGAGCACGTTC
>M_allele
TAGCACATAATTAGCTATACTACTACTACTACTACTACTACTACTACTACTACTACTACT
ACTACTACTACTACTACTACTACTACTACTACTACTACTACTACTACTACTACTACTAGT
AGTAGTAGTAGTAGTAGGAGTACTAGTAGTAGTGGGCGGTACTACTAGTAGTAGCTACTA
CTAGTACTAATAGTACTATTAGTGGTGGTCTTACTACTACTAGTACTACTAGTAGCACCA
GTCGTAGTACTAGCAAAGTACAGTACTACTAGTTATTACTAGTAGTAGTACTACTACTAG
ACAAGTAATATAGCAGTACTAATAGTGGTAGTGAAATAAGTTTCTGCTATTCATTGTACA
ATCCTACATAGGCCCAATATCAATGACATACATTTAGGAAAATCAAAATAATGAAGATAC
AATGACACATACTATGGGAACAGAGGTAAGTACCACCTAAAGACACTTTTTATTATTGTA
CTCATAACCGATTCCAGATTCGTGCCACACGTTCCTATCTACCACCATACCGTACGTAGA
CCCACATCAAGTAAACAATTAATGGGGTCAAGTAAGAAACTTGAAAACTACCATTACCAA
CACAACCAGAGAAATGGCGTTTATTCTTGTTTTTGTTTCTGACGGCTTCTGCACAATGGT
AGCAACGTAGATTAAAAGAGGACTGAACCAATTGAATCGCTGATTGGACCCGGGAGCCTT
TTTTGTTCAAGACACTGGACAAGGCTTAAACGATTTTTGAGTAGGAGTATGTGCCTTTTT
TCTTCAATCACAGATCATGTTCAAGGAGGAAAGAATAATACTATGAGTAATATTGTGAAT
AGAACCACGATAAAAACGAATACAACATCACAGCACAAGGCATGGCATTGTACTCAGCAT
AGCACAACACACAGAACGACACATAAAAGCCATTTCACTACACTtgtaAGCTTCGAAATC
AATCAATACAATTCATCGCCGATTGTTGTACATCATCATTTGTCTACTCCATATTACAAG
```

CAAGTGACAAAAAGAAAAAAAGATCTTTAGTCAAAATGAATGACGAATCGAATAAAGAAT
GGACCTACTACTTCGGTCGAGGAGGCGCTTGCAACCAACAGCGCAAGCAATCTAAGGTGG
TCGGTTTGTTGCAGAAACTGGCTCTTCGCTATAACCAGTGCCATCGAGAAGAGAAAAGAC
TTTTTGCGAAATGCGAAGTCTATAATATAGTTCTTGAAAAGGGAGGTTCATTTTTCGAAA
TAACGAACAAAATAGCCGTCGACGTCACGGCGGACGAAATGAAATCAACTACAAAGATCA
TGCAAGTTTTTCGAGATATAAATAAACGTAACAAGAAGAGTAAAGGGTCTGCAAAGGCGA
AAAAGAAGCCATCTTTCACTTCTCGAACGAAAAAAAATAAGCCGTCTTTTTGCACTTTAC
AAACTACAAGAGCAAGAGAATCACGAACGACCAAAATGAAAATAAAAACTCAACCAAAAC
GCAGGTGCGTCAAGCCTAGCATAATTGAAGCGCCAATCATGAATTGTGTGAGGGATGCTT
TACTGATGGCCAGCGCGAAAAGTCAATCGCTTCCTGACACAAGGACAAACAAGCTGGGGT
TGGGTACTCCGGTCAGGGGTAACGGATCCGAGCTGAAGCGACTTATTGCGGAAAGTAATC
ATCGCTTCTCAACAAATAATGAAACATTTGATTTAATGGCCGTGGAACCACCTCAGCTAG
AGAACTCGTTTAGTGCACTGATCATGGCGGATGAGGTAACAACGGAACTTCCAACTGATT
CTCAAATGAGTGTGAACCTACACCTGCACAAAAGAGTACAGCGACTAGAAAATCTTGTTG
GGATGCTAATGCAGCAGATGTAGAGAGTCTAGTGTAGCAAAATACTTAAACATGGAAACA
GGAATCACTCGACGCTACGTGTACCAAAAAATATGCAGCGATCGGATTTGCGCAACACAA
TCTGCTCTCTGGGCTAGCATCAAATTCCAATGAAAATGTTCCGCCCGATGGTGACTACTT
TGACTAAAAAACTTGCTTGAACTCCTGTTACAGCAAAATCAGATGCGCGTGAAAATATTT
GGTCAGCAAGAGCACGCAAAACTATTTGGTTGCAAGGAATAAAATTTGAGCACGTTC
>B_allele
GTGAATTGTAATACGACTCACTATAGGGCGAATTGGGCCCTCTAGATGCATGCTCGAGCG
GCCGCCAGTGTGATGGATATCTGCAGAATTCGCCCTTGGCAACAAGTGCTTGCTAATGCT
GCTGCCAAAGCTCAGACAACAGCACACTAGCAACAATAATTAGCTATACTACTAGTAGTA
GTAGTAGTAGTAGTAGTAGTAGTAGGAGGAGGAGGAGGAGGAGGAGgGAGTACTAGTA
GTAGTGGGCggtactactagtagtaGCTACTACTAGTACTAATAGTACTATTAGTGGTGG
TCTTACTACTACTAGTACTAGTAGCACCAGTCGTAGTACTAGCAAAGTACAGTACTACTA
GTTATTACTAGTAGTAGTACTACTACTAGACAAGTAATATAGCAGTACTAATGGTGGTAG
TGAAATAAGTTTCTGCTATTCATTGTACAATCCTACATAGGCCCAATATCAATGACATAC
ATTTAGGAAAATCAAAATAATGAAGATACAATGACACATACTATGGGAACAGAGGTAAGT
ACCACCTAAAGACACTTTTTATTATTGTACTCATAACCGATTCCAGATTCGTGCCACACG
TTCCTATCTACCACCATACCGTACGTAGACCCACATCAAGTAAACAATTAATGGGGTCAA
GTAAGAAACTTGAAAACTACCATTACCAACACAACCAGAGAAATGGCGTTTATCCTTGTT
TTTGTTTCTGACGGCTTCTGCACAATGGTAGCAACGTAGATTAAAAGAGGACTGAACCAA
TTGAATCGCTGATTGGACCCGGGAGCCTTTTTTGTTCAAGACACTGGACAAGGCTTAAAC
AATTTTTGAGTAGGAGTATGTGCCTTTTTTCTTCGATCACAGATCATGTTCAAGGAGGAA
AGAATAATACTACGAGTAATATTGTGAATAGAACCACAATAAAAACGAATACAACATCAC
AGCACAAGGCATGGCATTGTACTCAGCATAGCACAACACACATAACAACACATAAAAGCC
AtttcactacacttgtaAGCTTCGAAATCAATCAATACAATTCATCGCCGATTGTTGTAC
ATCATCATTTGTCTACTCCATATTACAAGCAAGTGACAAAAAGAAAAAAAGATCTTTAGT
CAAAATGAATGACGAATCGAATAAAGAATGGACCTACTACTTTGGTCGAGGAGGCGCTTG
CAACCAACAGCGCAAGCAATCTAAGGTGGTCGGTTTGTTGCAGAAACTGGCTCTTCGCTA
TAACCAGTGCCATCGAGAAGAGAAAAGACTTTTTGCGAAATGCGAAGTCTATAATATAGT
TCTTGAAAAGGGAGGTTCGTTTTTCGAAATAACGAACAAAATAGCCGTCGACGTCACGGC
GGACGAAATGAAATCAACTACAAAGATCATGCAAGTTTTTCGAGATATAAATAAATGTAA
CAAGAAGAGTAAAGGGTCTGCAAAGGCGAAAAAGAAGCCATCTTTCACTTCTCGAACGAA
AAAAAATAAGCTGTCTTTTTGCACTTTACAAACTACAAGAGCAAGAGAATCACGAACGAC
CAAAATGAAAATAAAAACTCAACCAAAACGCAGGTGCGTCAAGCCTAGCATAATTGAAGC
GCCAATCATGAATTGTGTGAGGGATGCTTTACTGATGGCCAGCGCGAAAAGTCAATCGCT
TCCTGACACAAGGACAAACAAGCCGGGGTTGGGTACTCCGGTCAGGGGTAACGGATCCGA
GCTGAAGCGACTTATTGCGGAAAGTAATCATCGCTTCTCAACAAATAATGAAACATTTGA
TTTAATGGCCGTGGAACCACCTCAGCTAGAGAACTCGTTTAGTGCACTGATCATGGCGGA
TGAGGTAACAACGGAACTTCCAACTGATTCTCAAATGAGTGTGAACCTACACCTGCACAA
AAGAGTACAGCGACTAGAAAATCTTGTTGGGATGCTAATGCAGCAGATGTAGAGAGTCTA
GTGTAGCTAAATACTTAAACATGGAAACAGGAATCACTCGACGCTACGTGTACCAAAAAA
TATGCAGCGATCGGATTTGCGCAACACAATCTGCTCTCTGGGCTAGCATCAAATTCCAAT
GAAAATGTTCCGCCCGATGGTGACTACTTTGACTAAAAAACTTGCTTGAACTCCTGTTAC

```
AGCAAAATCAGATGCGCGTGAAAATATTTGGTCAGCAAGAGCACGCAAAACTATTTGGTT
GCAAGGAATAAAATTTGAGCACGTTC
>N_allele
ATGACACATACTATGGGAACAGAGGtAAGTACCACCTAAAGACACTTtTTATTATTGTAC
TCATAACtGATTCCAGATTCGTGCCACACGTTCCTATCTACCACCATACCGTACGTAGAC
CCACATCAAGTAAACAATTAATGGGGTCAAGTAAGAAACTTGAAAACTACCATTACCAAC
ACAACCAGAGAAATGGCGTTTATTCTTGTTTTTGTTTCTGACGGCTTCTGCACAATGGTA
GCAACGTAGATTAAAAGAGGACTGAACCAATTGAATCGCTGATTGGACCCGGGAGCCTTT
TTTGTTCAAGACACTGGACAAGGCTTAAACGATTTTTGAGTAGGAGTATGTGCCTTTTTT
CTTCaATCACAGATCATGTTCAAGGAGGAAAGAATAATACTACGAGTAATATTGTGAATA
GAACCACGATAAAAACGAATACAACATCACAGCACAAGGCATGGCATTGTACTCAGCATA
GCACAACACACAGAACAACACATAAAAGCCATTTCACTACACTTGTAAGCTtCGAAATCA
ATCAaTACAATTCATCGCCGATTGTTGTACATCATCATTTGTCTACTCCATATTACAAgC
AAGTGACAAAAAGAAAAAAAGATCTTTAGTCAAAATGAATGACGAATCaAATAAAGAATG
GACCCACTACTTCGGTCGAGGAGGCGCTTGCAACCAACAGCGCAAGCAATCTAAGGTGGc
CGGTTTGTTGCAGAAACTGGCTCTTTGCTATAACCAGTGCCATCGAGAAGAGAAAAGACT
TTTTGCGAAATGCGAAGTCTATAATATAGTTCTTGAAAAGGGAGGTTCATTTTTCGAAAT
AACGAACAAAATAGCCGTCGACGTCACGGCGGACGAAATGAAATCAACTACAAAGATCAT
GCAAGTTTTTCGAGATATAAATAAACGTAACAAGAAGAGTAAAGGGTCTGCAAAGGCGAA
AAAGAAGCCATCTTTCACTTCTCGAACGAAAAAAAATAAGCCGTCTTTTTGCACTTTACA
AACTACAAGAGCAAGAGAATCACGAACGACCAAAATGAAAATAAAAACTCAACCAAAACG
CAGGTGCGTCAAGCCTAGCATAATTGAAGCGCCAATCATGAATTGTGTGAGGGATGCTTT
ACTGATGGCCAGCGCGAAAAGTCAATCGCTTCCTGACACAAGGACAAACAAGCTGGGGTT
GGGTACTCCGGTCAGGGGTAACGGATCCGAGCTGAAGCGACTTATTGCGGAAAGTAATCA
TCGCTTCTCAACAAATAATGAAACATTTGATTTAATGGCCGTGGAACCACCTCAGCTAGA
GAACTCGTTTAGTGCACTGATCATGGCGGATGAGGTAACAACGGAACTTCCAACTGATTC
TCAAATGAGTGTGAACCTACACCTGCACAAAAGAGTACAGCGACTAGAAAATCTTGTTGG
GATGCTAATGCAGCAGATGTAGAGAGTCTAGTGTAGCAAAATACTTAAACATGGAAACAG
GAATCACTCGACGCTACGTGTACCAAAAAATATGCAGCGATCGGATTTGCGCAACACAAT
CTGCTCTCTGGGCTAGCATCAAATTCCAATGAAAATGTTCCGCCCGATGGTGACTACTTT
GACTAAAAAACTTGCTTGAACTCCTGTTACAGCAAAATCAGATGCGCGTGAAAATATTTG
GTCAGCAAGAGCACGCAAAACTATTTGGTTGCAAGGAATAAAATTTGAGCACGTTC
```

Supplementary Figure 5. Sequences of the *MRP3* alleles. Nucleotide sequences of the A, M, B and N alleles.

```
A_allele      --------------------------------------AATAATTAGCTATACTACTACT  22
M_allele      ------------------------------------------------------------  0
B_allele      GTGAATTGTAATACGACTCACTATAGGGCGAATTGGGCCCTCTAGATGCATGCTCGAGCG  60
N_allele      ------------------------------------------------------------  0

A_allele      ACTACTACTACTACTACT------ACTACTACTACTACTACTACTACTACTACTACTACT  76
M_allele      ------------------TAGCACATAATTAGCTATACT---------ACTACTACTACT  33
B_allele      GCCGCCAGTGTGATGGATATCTGCAGAATTCGCCCTTGGCAACAAGTGCTTGCTAATGCT  120
N_allele      ------------------------------------------------------------  0

A_allele      ACTACTACTACTACTACTACTACTACTACTACTACTACTACTACTACTACTACTACTACT  136
M_allele      ACTACTACTACTAC------TACTACTACTACTACTACTACTACTACTACTACTACTACT  87
B_allele      GCTGCCAAAGCTCAG-ACAACAGCACACTAGCAACAATAATTAGCTATACTACTAGTAGT  179
N_allele      ------------------------------------------------------------  0

A_allele      ACTACTACTACTACTACTACTACTACTACTACTACTACTACTACTACTAGGAGTACTAGT  196
M_allele      ACTACTACTACTACTACTACTACTACTACTACTAGTAGTAGTAGTAGTAGGAGTACTAGT  147
B_allele      AGTAGTAGTAGTAGTAGTAGTAGTAGGAGGAGGAGGAGGAGGAGGAGGAgGAGTACTAGT  239
N_allele      ------------------------------------------------------------  0

A_allele      AGTAGTGGGCAGTACTACTAGTAGTAGCTACTAATAGTACTAATAGTACTATTAGTGGTG  256
M_allele      AGTAGTGGGCGGTACTACTAGTAGTAGCTACTACTAGTACTAATAGTACTATTAGTGGTG  207
B_allele      AGTAGTGGGCggtactactagtagtaGCTACTACTAGTACTAATAGTACTATTAGTGGTG  299
N_allele      ------------------------------------------------------------  0

A_allele      GTCTTACTACTACTAGTACTAGTAGCAC---CAGTCGTAGTACTAGCAAAGTACAGCAGT  313
M_allele      GTCTTACTACTACTAGTACTACTAGTAGCACCAGTCGTAGTACTAGCAAAGTAC------  261
B_allele      GTCTTACTACTACT---AGTACTAGTAGCACCAGTCGTAGTACTAGCAAAGTAC------  350
N_allele      ------------------------------------------------------------  0

A_allele      ACTACTACTAGTTATTACTAGTAGTACTAGTAGTACTAGACAAGTAATATAGCAGTACTA  373
M_allele      AGTACTACTAGTTATTACTAGTAGTAGTACTACTACTAGACAAGTAATATAGCAGTACTA  321
B_allele      AGTACTACTAGTTATTACTAGTAGTAGTACTACTACTAGACAAGTAATATAGCAGTACTA  410
N_allele      ------------------------------------------------------------  0

A_allele      ATAGTGGTAGTGAAATAAGTTTCTGCTATTCATTGTACAATCCTACATAGGCCCAATATC  433
M_allele      ATAGTGGTAGTGAAATAAGTTTCTGCTATTCATTGTACAATCCTACATAGGCCCAATATC  381
B_allele      ATGGTGGTAGTGAAATAAGTTTCTGCTATTCATTGTACAATCCTACATAGGCCCAATATC  470
N_allele      ------------------------------------------------------------  0

A_allele      AATGACAAACATTTAGGAAAATCAAAATAATGAAGATACAATGACACATACTATGGGAAC  493
M_allele      AATGACATACATTTAGGAAAATCAAAATAATGAAGATACAATGACACATACTATGGGAAC  441
B_allele      AATGACATACATTTAGGAAAATCAAAATAATGAAGATACAATGACACATACTATGGGAAC  530
N_allele      ----------------------------------------ATGACACATACTATGGGAAC  20
                                                      ********************

A_allele      AGAGGCAAGTACCACCTAAAGACACTTGTTATTATTGTACTCATAACCGATTCCAGATTC  553
M_allele      AGAGGTAAGTACCACCTAAAGACACTTTTTATTATTGTACTCATAACCGATTCCAGATTC  501
B_allele      AGAGGTAAGTACCACCTAAAGACACTTTTTATTATTGTACTCATAACCGATTCCAGATTC  590
N_allele      AGAGGtAAGTACCACCTAAAGACACTTtTTATTATTGTACTCATAACtGATTCCAGATTC  80
              ***** ********************* ******************* ************
```

```
A_allele      GTGCCACACGTTCCTATCTACCACCATACCGTACGTAGACCCACATCAAGTAAACAATTA  613
M_allele      GTGCCACACGTTCCTATCTACCACCATACCGTACGTAGACCCACATCAAGTAAACAATTA  561
B_allele      GTGCCACACGTTCCTATCTACCACCATACCGTACGTAGACCCACATCAAGTAAACAATTA  650
N_allele      GTGCCACACGTTCCTATCTACCACCATACCGTACGTAGACCCACATCAAGTAAACAATTA  140
              ************************************************************

A_allele      ATGGGGTCAAGTAAGAAACTTGAAAACTACCATTACCAACACAACCAGAGAAATGGCGTT  673
M_allele      ATGGGGTCAAGTAAGAAACTTGAAAACTACCATTACCAACACAACCAGAGAAATGGCGTT  621
B_allele      ATGGGGTCAAGTAAGAAACTTGAAAACTACCATTACCAACACAACCAGAGAAATGGCGTT  710
N_allele      ATGGGGTCAAGTAAGAAACTTGAAAACTACCATTACCAACACAACCAGAGAAATGGCGTT  200
              ************************************************************

A_allele      TATTCTTGTTTTTGTTTCTGACGGCTTCTGCACAATGGTAGCAACGTAGATTAAAAGAGG  733
M_allele      TATTCTTGTTTTTGTTTCTGACGGCTTCTGCACAATGGTAGCAACGTAGATTAAAAGAGG  681
B_allele      TATCCTTGTTTTTGTTTCTGACGGCTTCTGCACAATGGTAGCAACGTAGATTAAAAGAGG  770
N_allele      TATTCTTGTTTTTGTTTCTGACGGCTTCTGCACAATGGTAGCAACGTAGATTAAAAGAGG  260
              *** ********************************************************

A_allele      ACTGAACCAATTGAATCGCTGATTGGACCCGGGAGCCTTTTTTGTTCAAGACACTGGACA  793
M_allele      ACTGAACCAATTGAATCGCTGATTGGACCCGGGAGCCTTTTTTGTTCAAGACACTGGACA  741
B_allele      ACTGAACCAATTGAATCGCTGATTGGACCCGGGAGCCTTTTTTGTTCAAGACACTGGACA  830
N_allele      ACTGAACCAATTGAATCGCTGATTGGACCCGGGAGCCTTTTTTGTTCAAGACACTGGACA  320
              ************************************************************

A_allele      AGGCTTAAACGATTTTTGAGTAGGAGTATGTGCCTTTTTTCTTCGATCACAGATCATGTT  853
M_allele      AGGCTTAAACGATTTTTGAGTAGGAGTATGTGCCTTTTTTCTTCAATCACAGATCATGTT  801
B_allele      AGGCTTAAACAATTTTTGAGTAGGAGTATGTGCCTTTTTTCTTCGATCACAGATCATGTT  890
N_allele      AGGCTTAAACGATTTTTGAGTAGGAGTATGTGCCTTTTTTCTTCaATCACAGATCATGTT  380
              ********** ********************************* ***************

A_allele      CAAGGAGGAAAGAATAATACTACGAGTAATATTGTGAATAGAACCACGATAAAAACGAAT  913
M_allele      CAAGGAGGAAAGAATAATACTATGAGTAATATTGTGAATAGAACCACGATAAAAACGAAT  861
B_allele      CAAGGAGGAAAGAATAATACTACGAGTAATATTGTGAATAGAACCACAATAAAAACGAAT  950
N_allele      CAAGGAGGAAAGAATAATACTACGAGTAATATTGTGAATAGAACCACGATAAAAACGAAT  440
              ********************** ************************ ************

A_allele      ACAACATCACAGCACAAGGCATGGCATTGTACTCAGCATAGCACAACACACCGAACAACA  973
M_allele      ACAACATCACAGCACAAGGCATGGCATTGTACTCAGCATAGCACAACACACAGAACGACA  921
B_allele      ACAACATCACAGCACAAGGCATGGCATTGTACTCAGCATAGCACAACACACATAACAACA  1010
N_allele      ACAACATCACAGCACAAGGCATGGCATTGTACTCAGCATAGCACAACACACAGAACAACA  500
              ***************************************************  *** ***

A_allele      CATAAAAGCCATTTCACTACACTtgtaAGCTCCGAAATCAATCAGTACAATTCATCGCCG  1033
M_allele      CATAAAAGCCATTTCACTACACTtgtaAGCTTCGAAATCAATCAATACAATTCATCGCCG  981
B_allele      CATAAAAGCCAtttcactacacttgtaAGCTTCGAAATCAATCAATACAATTCATCGCCG  1070
N_allele      CATAAAAGCCATTTCACTACACTTGTAAGCTTCGAAATCAATCAATACAATTCATCGCCG  560
              ******************************* ************ ***************

A_allele      ATTGTTGTACATCATCATTTGTCTACTCCATATTACAAACAAGTGACAAAAAGAAAAAAA  1093
M_allele      ATTGTTGTACATCATCATTTGTCTACTCCATATTACAAGCAAGTGACAAAAAGAAAAAAA  1041
B_allele      ATTGTTGTACATCATCATTTGTCTACTCCATATTACAAGCAAGTGACAAAAAGAAAAAAA  1130
N_allele      ATTGTTGTACATCATCATTTGTCTACTCCATATTACAAGCAAGTGACAAAAAGAAAAAAA  620
              ************************************** *********************

A_allele      GATCTTTAGTCAAAATGAATGACGAATCGAATAAAGAATGGACCTACTACTTCGGTCGAG  1153
M_allele      GATCTTTAGTCAAAATGAATGACGAATCGAATAAAGAATGGACCTACTACTTCGGTCGAG  1101
B_allele      GATCTTTAGTCAAAATGAATGACGAATCGAATAAAGAATGGACCTACTACTTTGGTCGAG  1190
N_allele      GATCTTTAGTCAAAATGAATGACGAATCAAATAAAGAATGGACCCACTACTTCGGTCGAG  680
              *************************** **************** ******* *******

A_allele      GAGGCGCTTGCAACCAACAGCGCAAGCAATCTAAGGTGGTCGGTTTGTTGCAGAAACTGG  1213
M_allele      GAGGCGCTTGCAACCAACAGCGCAAGCAATCTAAGGTGGTCGGTTTGTTGCAGAAACTGG  1161
B_allele      GAGGCGCTTGCAACCAACAGCGCAAGCAATCTAAGGTGGTCGGTTTGTTGCAGAAACTGG  1250
```

```
N_allele      GAGGCGCTTGCAACCAACAGCGCAAGCAATCTAAGGTGGCCGGTTTGTTGCAGAAACTGG   740
              *************************************** ********************

A_allele      CTCTCCGCTATAACCAGTGCCATCGAGAAGAGAAAAGACTTTTTGCGAAATGCGAAGTCT   1273
M_allele      CTCTTCGCTATAACCAGTGCCATCGAGAAGAGAAAAGACTTTTTGCGAAATGCGAAGTCT   1221
B_allele      CTCTTCGCTATAACCAGTGCCATCGAGAAGAGAAAAGACTTTTTGCGAAATGCGAAGTCT   1310
N_allele      CTCTTTGCTATAACCAGTGCCATCGAGAAGAGAAAAGACTTTTTGCGAAATGCGAAGTCT   800
              ****  ******************************************************

A_allele      ATAATATAGTTCTTGAAAAGGGAGGTTCGTTTTTCGAAATAACGAACAAAATAGCCGTCG   1333
M_allele      ATAATATAGTTCTTGAAAAGGGAGGTTCATTTTTCGAAATAACGAACAAAATAGCCGTCG   1281
B_allele      ATAATATAGTTCTTGAAAAGGGAGGTTCGTTTTTCGAAATAACGAACAAAATAGCCGTCG   1370
N_allele      ATAATATAGTTCTTGAAAAGGGAGGTTCATTTTTCGAAATAACGAACAAAATAGCCGTCG   860
              **************************** *******************************

A_allele      ACGTCACGGCGGACGAAATGAAATCAACTACAAAGATCATGCAAGTTTTTCGAGATATAA   1393
M_allele      ACGTCACGGCGGACGAAATGAAATCAACTACAAAGATCATGCAAGTTTTTCGAGATATAA   1341
B_allele      ACGTCACGGCGGACGAAATGAAATCAACTACAAAGATCATGCAAGTTTTTCGAGATATAA   1430
N_allele      ACGTCACGGCGGACGAAATGAAATCAACTACAAAGATCATGCAAGTTTTTCGAGATATAA   920
              ************************************************************

A_allele      ATAAACGTAACAAGAAGAGTAAAGGGTCTGCAAAGGCGAAAAAGAAGCCATCTTTCACTT   1453
M_allele      ATAAACGTAACAAGAAGAGTAAAGGGTCTGCAAAGGCGAAAAAGAAGCCATCTTTCACTT   1401
B_allele      ATAAATGTAACAAGAAGAGTAAAGGGTCTGCAAAGGCGAAAAAGAAGCCATCTTTCACTT   1490
N_allele      ATAAACGTAACAAGAAGAGTAAAGGGTCTGCAAAGGCGAAAAAGAAGCCATCTTTCACTT   980
              ***** ******************************************************

A_allele      CTCGAACGAAAAAAAATAAGCCGTCTTTTTGCACTTTACAAACTACTAGAGCAAGAGAAT   1513
M_allele      CTCGAACGAAAAAAAATAAGCCGTCTTTTTGCACTTTACAAACTACAAGAGCAAGAGAAT   1461
B_allele      CTCGAACGAAAAAAAATAAGCTGTCTTTTTGCACTTTACAAACTACAAGAGCAAGAGAAT   1550
N_allele      CTCGAACGAAAAAAAATAAGCCGTCTTTTTGCACTTTACAAACTACAAGAGCAAGAGAAT   1040
              ********************* ************************ *************

A_allele      CACGAACGACCAAAATGAAAATAAAAACTCAACCAAAACGCAGGTGCGTCAAGCCTAGCA   1573
M_allele      CACGAACGACCAAAATGAAAATAAAAACTCAACCAAAACGCAGGTGCGTCAAGCCTAGCA   1521
B_allele      CACGAACGACCAAAATGAAAATAAAAACTCAACCAAAACGCAGGTGCGTCAAGCCTAGCA   1610
N_allele      CACGAACGACCAAAATGAAAATAAAAACTCAACCAAAACGCAGGTGCGTCAAGCCTAGCA   1100
              ************************************************************

A_allele      TAATTGAAGCGCCAATCATGAATTGTGTGAGGGATGCTTTACTGATGGCCAGCGCGAAAA   1633
M_allele      TAATTGAAGCGCCAATCATGAATTGTGTGAGGGATGCTTTACTGATGGCCAGCGCGAAAA   1581
B_allele      TAATTGAAGCGCCAATCATGAATTGTGTGAGGGATGCTTTACTGATGGCCAGCGCGAAAA   1670
N_allele      TAATTGAAGCGCCAATCATGAATTGTGTGAGGGATGCTTTACTGATGGCCAGCGCGAAAA   1160
              ************************************************************

A_allele      GTCAATCGCTTCCTGACACAAGGACAAACAAGCCGGGGTTGGGTACTCCGGTCAGGGGTA   1693
M_allele      GTCAATCGCTTCCTGACACAAGGACAAACAAGCTGGGGTTGGGTACTCCGGTCAGGGGTA   1641
B_allele      GTCAATCGCTTCCTGACACAAGGACAAACAAGCCGGGGTTGGGTACTCCGGTCAGGGGTA   1730
N_allele      GTCAATCGCTTCCTGACACAAGGACAAACAAGCTGGGGTTGGGTACTCCGGTCAGGGGTA   1220
              ********************************* **************************

A_allele      ACGGATCCGAGCTGAAGCGACTTATTGCGGAAAGTAATCATCGCTTCTCAACAAATAATG   1753
M_allele      ACGGATCCGAGCTGAAGCGACTTATTGCGGAAAGTAATCATCGCTTCTCAACAAATAATG   1701
B_allele      ACGGATCCGAGCTGAAGCGACTTATTGCGGAAAGTAATCATCGCTTCTCAACAAATAATG   1790
N_allele      ACGGATCCGAGCTGAAGCGACTTATTGCGGAAAGTAATCATCGCTTCTCAACAAATAATG   1280
              ************************************************************

A_allele      AAACATTTGATTTAATGGCCGTGGAACCACCTCAGCTAGAGAACTCGTTTAGTGCACTGA   1813
M_allele      AAACATTTGATTTAATGGCCGTGGAACCACCTCAGCTAGAGAACTCGTTTAGTGCACTGA   1761
B_allele      AAACATTTGATTTAATGGCCGTGGAACCACCTCAGCTAGAGAACTCGTTTAGTGCACTGA   1850
N_allele      AAACATTTGATTTAATGGCCGTGGAACCACCTCAGCTAGAGAACTCGTTTAGTGCACTGA   1340
              ************************************************************
```

```
A_allele      TCATGGCGGATGAGGTAACAACGGAACTTCCAACTGATTCTCAAATGAGTGTGAACCTAC  1873
M_allele      TCATGGCGGATGAGGTAACAACGGAACTTCCAACTGATTCTCAAATGAGTGTGAACCTAC  1821
B_allele      TCATGGCGGATGAGGTAACAACGGAACTTCCAACTGATTCTCAAATGAGTGTGAACCTAC  1910
N_allele      TCATGGCGGATGAGGTAACAACGGAACTTCCAACTGATTCTCAAATGAGTGTGAACCTAC  1400
              ************************************************************

A_allele      ACCTGCACAAAAGAGTACAGCGACTAGAAAATCTTGTTGGGATGCTAATGCAGCAGATGt  1933
M_allele      ACCTGCACAAAAGAGTACAGCGACTAGAAAATCTTGTTGGGATGCTAATGCAGCAGATGT  1881
B_allele      ACCTGCACAAAAGAGTACAGCGACTAGAAAATCTTGTTGGGATGCTAATGCAGCAGATGT  1970
N_allele      ACCTGCACAAAAGAGTACAGCGACTAGAAAATCTTGTTGGGATGCTAATGCAGCAGATGT  1460
              ************************************************************

A_allele      agAGAGTCTAGTGTAGCTAAATACTTAAACATGGAAACAGGAATCACTCGACGCTACGTG  1993
M_allele      AGAGAGTCTAGTGTAGCAAAATACTTAAACATGGAAACAGGAATCACTCGACGCTACGTG  1941
B_allele      AGAGAGTCTAGTGTAGCTAAATACTTAAACATGGAAACAGGAATCACTCGACGCTACGTG  2030
N_allele      AGAGAGTCTAGTGTAGCAAAATACTTAAACATGGAAACAGGAATCACTCGACGCTACGTG  1520
              ***************** ******************************************

A_allele      TATCAAAAAATATGCAGCGATCGGATTTGCGCAACACAATCTGCTCTCTGGGCTGGCATC  2053
M_allele      TACCAAAAAATATGCAGCGATCGGATTTGCGCAACACAATCTGCTCTCTGGGCTAGCATC  2001
B_allele      TACCAAAAAATATGCAGCGATCGGATTTGCGCAACACAATCTGCTCTCTGGGCTAGCATC  2090
N_allele      TACCAAAAAATATGCAGCGATCGGATTTGCGCAACACAATCTGCTCTCTGGGCTAGCATC  1580
              ** *************************************************** *****

A_allele      AAATTCCAAGGAAAATGTTCCGCCCGATGGTGACTACTTTGACTAAAAAACTTGCTTGAA  2113
M_allele      AAATTCCAATGAAAATGTTCCGCCCGATGGTGACTACTTTGACTAAAAAACTTGCTTGAA  2061
B_allele      AAATTCCAATGAAAATGTTCCGCCCGATGGTGACTACTTTGACTAAAAAACTTGCTTGAA  2150
N_allele      AAATTCCAATGAAAATGTTCCGCCCGATGGTGACTACTTTGACTAAAAAACTTGCTTGAA  1640
              ********* **************************************************

A_allele      CTCCTGTTACAGCAAAATCAGATGCGCGTGAAAATATTTGGTCAGCAAGAGCACGCAAAA  2173
M_allele      CTCCTGTTACAGCAAAATCAGATGCGCGTGAAAATATTTGGTCAGCAAGAGCACGCAAAA  2121
B_allele      CTCCTGTTACAGCAAAATCAGATGCGCGTGAAAATATTTGGTCAGCAAGAGCACGCAAAA  2210
N_allele      CTCCTGTTACAGCAAAATCAGATGCGCGTGAAAATATTTGGTCAGCAAGAGCACGCAAAA  1700
              ************************************************************

A_allele      CTATTTGGTTGCAAGGAATAAAATTTGAGCACGTTC 2209
M_allele      CTATTTGGTTGCAAGGAATAAAATTTGAGCACGTTC 2157
B_allele      CTATTTGGTTGCAAGGAATAAAATTTGAGCACGTTC 2246
N_allele      CTATTTGGTTGCAAGGAATAAAATTTGAGCACGTTC 1736
              ************************************
```

Supplementary Figure 6. Sequence alignment of the four *MRP3* alleles. Alignment of the A, M, B and N *MRP3* alleles. The putative transcription start site is in bold and underlined, in the transcribed portion of the gene nucleotide variants are indicated with colored letters. Asterisks indicate bases identical in all four sequences.

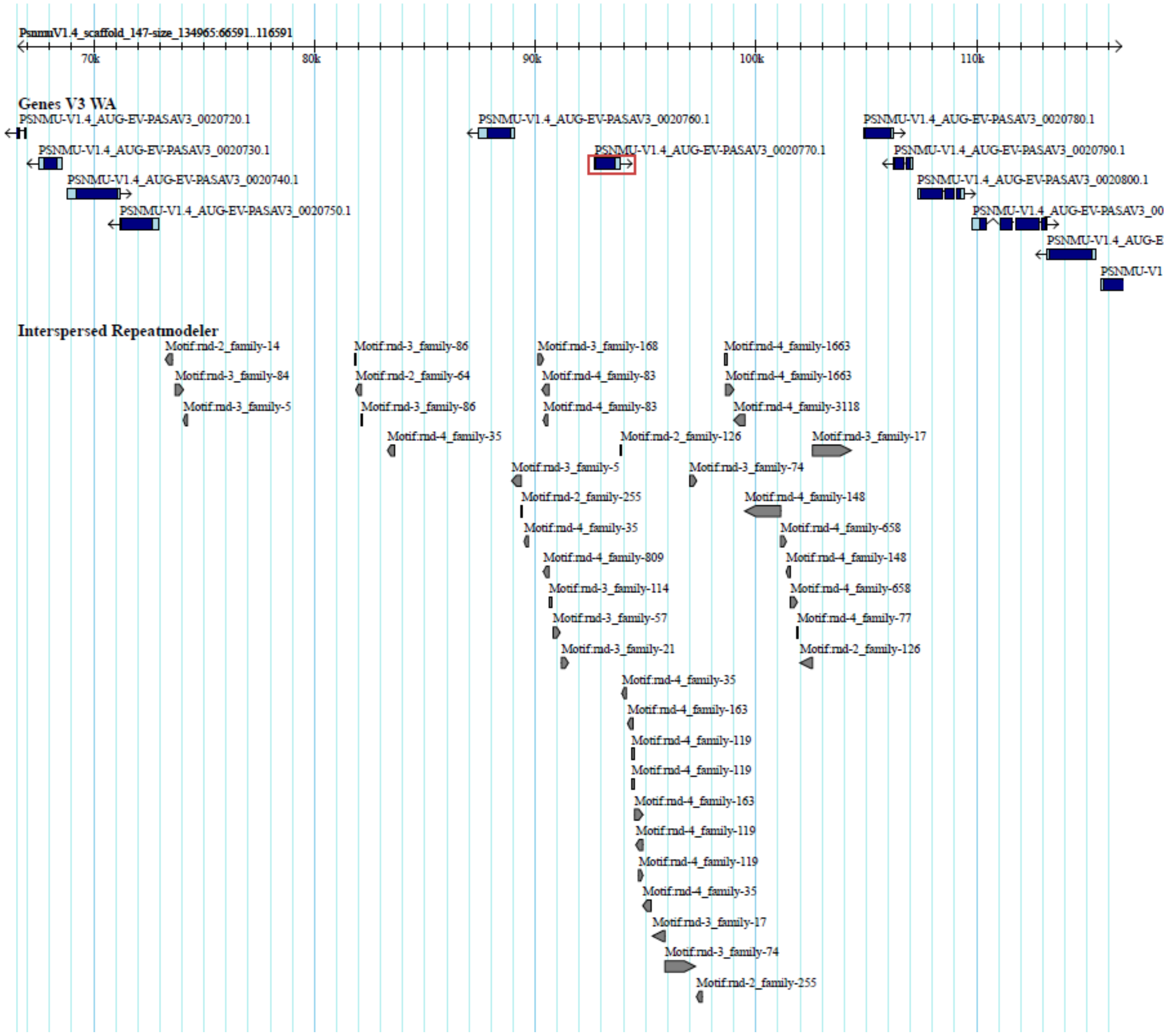

Supplementary Figure 7. ***MRP3* genomic landscape**. A portion of scaffold 147 is depicted. The genomic regions upstream and downstream of *MRP3*, indicated by a red rectangle, are rich in repetitive sequences (gray arrowheads in the lower track) and no coding genes can be found except for one gene located 4 kb upstream of *MRP3*.

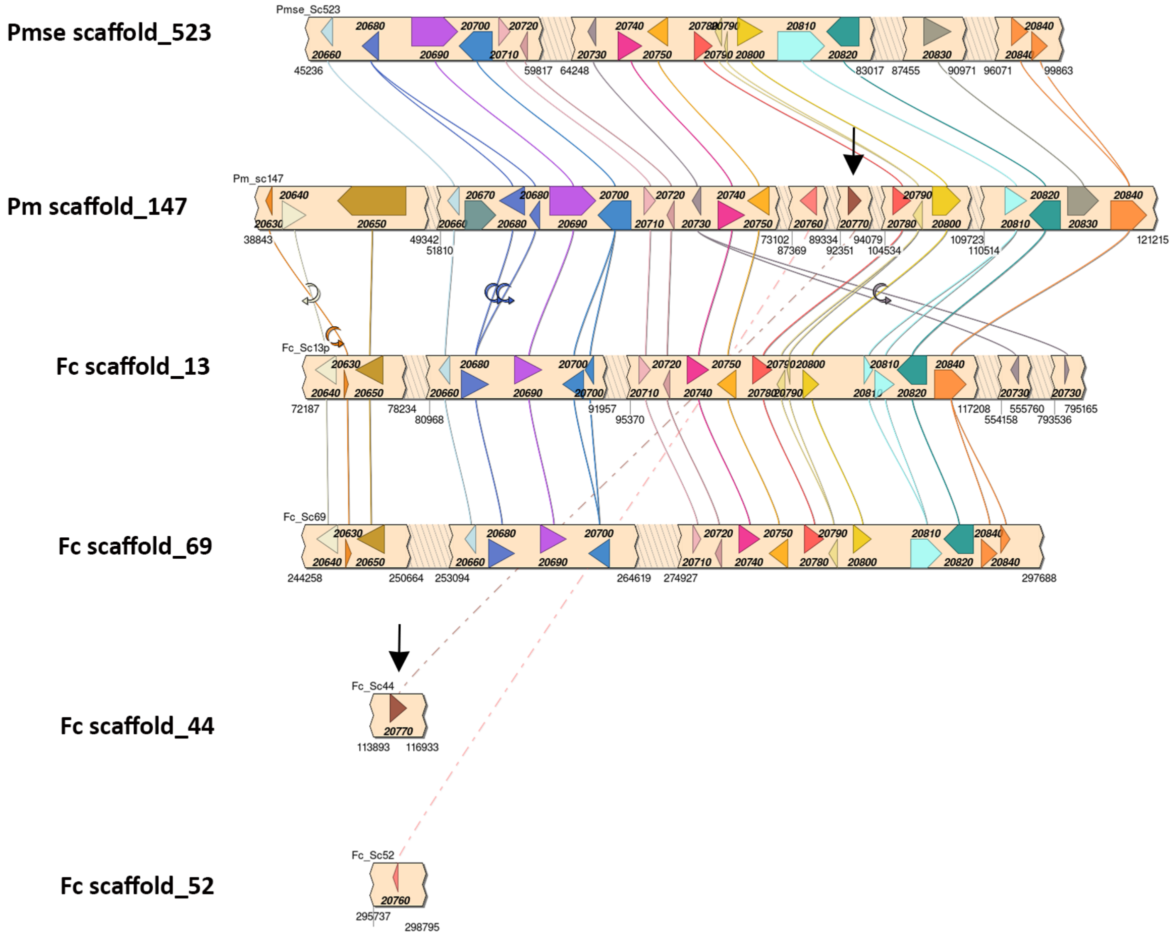

Supplementary Figure 8. Syntenic analysis of the *MRP3* genomic region. From the top, schematic representations of *Pseudo-nitzschia multiseries* (Pmse) scaffold 523, *Pseudo-nitzschia multistriata* (Pm) scaffold 147, *Fragilariopsis cylindrus* (Fc) scaffold 13, scaffold 69, scaffold 44 and scaffold 52. Scaffold segments are numbered with starting and ending nucleotide positions. Arrowheads represent genes. Homologous genes share a single color across species. *P. multistriata* gene names are indicated below the arrowheads, only the last five numbers of gene IDs are reported. The *MRP3* gene (ID 0020770) and its homologue in *F. cylindrus* are indicated by a vertical black arrow. No *MRP3* homologs can be found in the *Pseudo-nitzschia multiseries* genome. Synteny can be observed among the three species considered for genes upstream and downstream of *MRP3*, while *MRP3* itself and its neighboring gene 0020760 do not appear to retain synteny.

a

```
MRP3_A_Pm      MNDESNKEWTYYFGRGGACNQQRKQSKVVGLLQKLALRYNQCHREEKRLFAKCEVYNIVL  60
MRP3_M_Pm      MNDESNKEWTYYFGRGGACNQQRKQSKVVGLLQKLALRYNQCHREEKRLFAKCEVYNIVL  60
MRP3_B_Pm      MNDESNKEWTYYFGRGGACNQQRKQSKVVGLLQKLALRYNQCHREEKRLFAKCEVYNIVL  60
MRP3_N_Pm      MNDESNKEWTHYFGRGGACNQQRKQSKVAGLLQKLALCYNQCHREEKRLFAKCEVYNIVL  60
               **********:*****************.******** **********************

MRP3_A_Pm      EKGGSFFEITNKIAVDVTADEMKSTTKIMQVFRDINKRNKKSKGSAKAKKKPSFTSRTKK  120
MRP3_M_Pm      EKGGSFFEITNKIAVDVTADEMKSTTKIMQVFRDINKRNKKSKGSAKAKKKPSFTSRTKK  120
MRP3_B_Pm      EKGGSFFEITNKIAVDVTADEMKSTTKIMQVFRDINKCNKKSKGSAKAKKKPSFTSRTKK  120
MRP3_N_Pm      EKGGSFFEITNKIAVDVTADEMKSTTKIMQVFRDINKRNKKSKGSAKAKKKPSFTSRTKK  120
               ************************************ ***********************

MRP3_A_Pm      NKPSFCTLQTTRARESRTTKMKIKTQPKRRCVKPSIIEAPIMNCVRDALLMASAKSQSLP  180
MRP3_M_Pm      NKPSFCTLQTTRARESRTTKMKIKTQPKRRCVKPSIIEAPIMNCVRDALLMASAKSQSLP  180
MRP3_B_Pm      NKLSFCTLQTTRARESRTTKMKIKTQPKRRCVKPSIIEAPIMNCVRDALLMASAKSQSLP  180
MRP3_N_Pm      NKPSFCTLQTTRARESRTTKMKIKTQPKRRCVKPSIIEAPIMNCVRDALLMASAKSQSLP  180
               ** *********************************************************

MRP3_A_Pm      DTRTNKPGLGTPVRGNGSELKRLIAESNHRFSTNNETFDLMAVEPPQLENSFSALIMADE  240
MRP3_M_Pm      DTRTNKLGLGTPVRGNGSELKRLIAESNHRFSTNNETFDLMAVEPPQLENSFSALIMADE  240
MRP3_B_Pm      DTRTNKPGLGTPVRGNGSELKRLIAESNHRFSTNNETFDLMAVEPPQLENSFSALIMADE  240
MRP3_N_Pm      DTRTNKLGLGTPVRGNGSELKRLIAESNHRFSTNNETFDLMAVEPPQLENSFSALIMADE  240
               ****** *****************************************************

MRP3_A_Pm      VTTELPTDSQMSVNLHLHKRVQRLENLVGMLMQQM    275
MRP3_M_Pm      VTTELPTDSQMSVNLHLHKRVQRLENLVGMLMQQM    275
MRP3_B_Pm      VTTELPTDSQMSVNLHLHKRVQRLENLVGMLMQQM    275
MRP3_N_Pm      VTTELPTDSQMSVNLHLHKRVQRLENLVGMLMQQM    275
               ***********************************
```

b

```
CAMNT_0003586753_Pseudo-nitzschia_delicatissima          ------MSETSKSEWDFYFGRGGGSNKKRQVSKANILVHDLAFRYSY--CSQTEKRLFAK  52
TRINITY_DN7331_c0_g1_i1_Pseudo-nitzschia_arenysensis     ------MSDCSNSEWDFYFGRGGGSNQKRQVSKANSLVQDLAVRYSY--CRQTEKRLFAK  52
MRP3_Pseudo-nitzschia_multistriata                       ------MNDESNKEWTYYFGRGGACNQQRKQSKVVGLLQKLALRYNQ--CHREEKRLFAK  52
CAMNT_0008188179_Pseudo-nitzschia_australis              ------HPHQTDEEWNFYFGRGRSRNHQRKSSKVSGLLQNLAPRYRT--CDPGHRRLFAK  52
CAMNT_0047551615_Pseudo-nitzschia_fraudulenta            ------------------------------------------------------------  0
CAMNT_0013109375_Pseudo-nitzschia_pungens_cingulata      ------MTDTAREQWVFYFGRGGIGNKKRKCSKVSELLQKLAPRYST--CHPTERRLFAK  52
CAMNT_0013140303_Pseudo-nitzschia_pungens_pungens        ------------------------------------------------------------  0
jgi_Fracy1|272356_Fragilariopsis_cylindrus               ------------------------------------------------------------  0
CAMNT_0041255159_Fragilariopsis_kerguelensis_L2-C3       MMMMNNNSLNNNDEWNFYFGRGAENNKARKTSRANNLIQELAPLYIDPRTRPSDKQTFAK  60
CAMNT_0011363179_Fragilariopsis_kerguelensis_L26-C5      ------------------------------------------------------------  0


CAMNT_0003586753_Pseudo-nitzschia_delicatissima          NEVYDTVVKNGGTFFLVANKEIIDVTKDFDDTISRIMQSFRDINKSRRATSQTLSTNVEA  112
TRINITY_DN7331_c0_g1_i1_Pseudo-nitzschia_arenysensis     NEVYDTVVNNGGTFFLVTNKEFIDVTKDFDDTINRIMQSFRDINKICKTTSQPVSTRAEA  112
MRP3_Pseudo-nitzschia_multistriata                       CEVYNIVLEKGGSFFEITNKIAVDVTADEMKSTTKIMQVFRDINKRNKKSKGSAKAKKK-  111
CAMNT_0008188179_Pseudo-nitzschia_australis              HEVYNAVLKKGGSFFKLQNNMPVDVTADEFESTTKIMQAFRDIKKQCKLASTQSHLGSSH  112
CAMNT_0047551615_Pseudo-nitzschia_fraudulenta            --------------------------------MTKIMQAFRDINKQCRTPPRPSPS-LHP  27
CAMNT_0013109375_Pseudo-nitzschia_pungens_cingulata      NEVYNTVLNNGGAFFQIKDKLPVNVTANEHHSTTKIMQAFRDINKNGKNVPTHSHVGTHS  112
CAMNT_0013140303_Pseudo-nitzschia_pungens_pungens        ------------------------------------MQAFRDINKNGKNVPTHSHVDTHS  24
jgi_Fracy1|272356_Fragilariopsis_cylindrus               ------------------------------------------------------------  0
CAMNT_0041255159_Fragilariopsis_kerguelensis_L2-C3       QKVYDVVVNNGGRFIEKDKNI----TADKVACLKKIMQGLRDCNKVVNKQ--------CK  108
CAMNT_0011363179_Fragilariopsis_kerguelensis_L26-C5      ------------------------------------MQGLRDCNKVVNKQ--------CK  16


CAMNT_0003586753_Pseudo-nitzschia_delicatissima          KKVT-------------------------------N-----------------NKSMQST  124
TRINITY_DN7331_c0_g1_i1_Pseudo-nitzschia_arenysensis     KKVPSQKRSSPILMPT---------------ARDAN-----------------RKNMQST  140
MRP3_Pseudo-nitzschia_multistriata                       PSFT--------------------------SRTKKN------KP--------SFCTLQTT  131
CAMNT_0008188179_Pseudo-nitzschia_australis              LTTSSRK--NK--------------IKRM--KTSSSQ-----------KNKTKRPRSETP  143
CAMNT_0047551615_Pseudo-nitzschia_fraudulenta            PSSPRKK--RPLSTPGAGKKNSPPKKKAMTSRQSRNDSSME-RPSTMVHDVPSRLNRNHP  84
CAMNT_0013109375_Pseudo-nitzschia_pungens_cingulata      PSP--PR-----------------KTK---TKA----------P-----------KRKVQ  129
CAMNT_0013140303_Pseudo-nitzschia_pungens_pungens        PSPPPPR-----------------KTN---TKA----------P-----------KRKVQ  43
jgi_Fracy1|272356_Fragilariopsis_cylindrus               ------------------------------------------------------------  0
CAMNT_0041255159_Fragilariopsis_kerguelensis_L2-C3       PLPPSSP-----------------KSL---LKKTRDESRVKKTPSN------CLEIINVP  142
CAMNT_0011363179_Fragilariopsis_kerguelensis_L26-C5      PLPPSSP-----------------KSL---LKKTRDESRVKKTPSN------CLEIINVP  50


CAMNT_0003586753_Pseudo-nitzschia_delicatissima          SLNTKRVPSLKKSSRPKRRCIRPSVIEPPEIDCVRDTLRVIDTEKITPYTTTQL---HIG  181
TRINITY_DN7331_c0_g1_i1_Pseudo-nitzschia_arenysensis     SVKTKRVSLIKKSPRPKRRCIRPSIIEPPVIECVRDALRVVDADKIKQNGKTQI---SVG  197
MRP3_Pseudo-nitzschia_multistriata                       RARESRTTKMKIKTQPKRRCVKPSIIEAPIMNCVRDALLMASAKSQSLPDTRT-----NK  186
CAMNT_0008188179_Pseudo-nitzschia_australis              STKNPSPTMP--SSPPKPRCVKPSIIEPPFIDCVRDARRMVDVNESIPEISAVSSEMKPS  201
CAMNT_0047551615_Pseudo-nitzschia_fraudulenta            SIKRPSTTMHDVTSRPKRRCLKPSIIEPPITECVKEALRVVKMDKTKPRGNSTKAKKVVE  144
CAMNT_0013109375_Pseudo-nitzschia_pungens_cingulata      STKGPSPTMR--LSPAKRRCVKPSIIEPPILDCVRKALRMFNADESIPDVGSDRAERHCE  187
CAMNT_0013140303_Pseudo-nitzschia_pungens_pungens        STKRPSPTMR--LSPAKRRCVKPSIIEPPIMDCVREALRMFNVDESIPDVGSDRAERHCE  101
jgi_Fracy1|272356_Fragilariopsis_cylindrus               -----------------------------------------------MTSALK-----VD  8
CAMNT_0041255159_Fragilariopsis_kerguelensis_L2-C3       SLKRTSSIIP--VRCQKRACVKLPIVEAPIIECVRDALNVAKISGENQADTVN-----ID  195
CAMNT_0011363179_Fragilariopsis_kerguelensis_L26-C5      SLKRTSSIIP--VRCQKRACVKLPIVEAPIIECVRDALNVAKISGENQADTVN-----ID  103


CAMNT_0003586753_Pseudo-nitzschia_delicatissima          ELNILCDDG--ESRNTSINENGLCQLAADDELEKMVVEKLKLDNFFSQVVMTDEIKARHP  239
TRINITY_DN7331_c0_g1_i1_Pseudo-nitzschia_arenysensis     EIHTTSCDD--GTSKSKFDERGLCQLAADDAFEKMVVEPPKLDNSFSQLIMTDEIKA---  252
MRP3_Pseudo-nitzschia_multistriata                       XGLGTPVRGNGSELKRLIAESNHRFSTNNETFDLMAVEPPQLENSFSALIMADEVTT---  243
CAMNT_0008188179_Pseudo-nitzschia_australis              SNHKIPVSGGGSAQKPLVKKDTIRNLSTDDMFDTMAVEEPKLESSFSALIMADEITA---  258
CAMNT_0047551615_Pseudo-nitzschia_fraudulenta            RNV-LGGDGGERIEKSFDGKYGHHALTYDDPLARMAVEPPKLNDSFSALIMTDEITA---  200
CAMNT_0013109375_Pseudo-nitzschia_pungens_cingulata      NSNQASDVTCGQTPL---NKDSTRHFLSNDTFETMIVEPPKLENSFSALIMADEITA---  241
CAMNT_0013140303_Pseudo-nitzschia_pungens_pungens        NSNQASDVTCGQTPL---NKDSTRHFSSNDAFETMIVEPPKLENSFSALIMADEITA---  155
jgi_Fracy1|272356_Fragilariopsis_cylindrus               GENSLSGG-----GG---ARQELYGSEDNDVLERMAAEPPKLENSFSALIMADEITA---  57
CAMNT_0041255159_Fragilariopsis_kerguelensis_L2-C3       EKKAPSGI-----RV---AREEKCDNENKDILEIMALEPPQLENSFSALIMTDEITA---  244
CAMNT_0011363179_Fragilariopsis_kerguelensis_L26-C5      EKKAPSGI-----RV---AREEKCDNENKDILEIMALEPPQLENSFSALIMTDEITA---  152
                                                                            .        .: :  *  *  :*:. ** ::*:**:.:

CAMNT_0003586753_Pseudo-nitzschia_delicatissima          QQSLNLHLRVQRLENLVAMLMQIKSRLPTDAQRSANLNLHSRVQRLENLVSMVMQRKNDE  299
TRINITY_DN7331_c0_g1_i1_Pseudo-nitzschia_arenysensis     -------------------------HLPTDSQQSVNLNLHSRVQRLENLVAMLMQQKNDE  287
MRP3_Pseudo-nitzschia_multistriata                       -------------------------ELPTDSQMSVNLHLHKRVQRLENLVGMLMQQM---  275
CAMNT_0008188179_Pseudo-nitzschia_australis              -------------------------DLPTDSQKSINQNLHERVQRLENLVGMLMQQQNVD  293
CAMNT_0047551615_Pseudo-nitzschia_fraudulenta            -------------------------VLPSDSQKSINENLHSRIQRLENLVAMLMQQKNDE  235
CAMNT_0013109375_Pseudo-nitzschia_pungens_cingulata      -------------------------DLPTDSQKSVNQNLHKRVQRLENLVSMLMQQQNED  276
CAMNT_0013140303_Pseudo-nitzschia_pungens_pungens        -------------------------DLPTDSQKSVNQNLHKRVQRLENLVSMLMQQQNED  190
jgi_Fracy1|272356_Fragilariopsis_cylindrus               -------------------------DLPTDSQKNINQSLHARVQRLENLVAMLMQQKNED  92
CAMNT_0041255159_Fragilariopsis_kerguelensis_L2-C3       -------------------------DLPTGSLEEYKQSVHARVQRLENLVAMLMQQKNEE  279
CAMNT_0011363179_Fragilariopsis_kerguelensis_L26-C5      -------------------------DH----------VLLARVQRLENLVTMLMQQKNEE  177
                                                                                               :  *:******* *:**:

CAMNT_0003586753_Pseudo-nitzschia_delicatissima          KELKSAARIISNF     312
TRINITY_DN7331_c0_g1_i1_Pseudo-nitzschia_arenysensis     MELLLK-------     293
MRP3_Pseudo-nitzschia_multistriata                       -------------     275
CAMNT_0008188179_Pseudo-nitzschia_australis              LERLL--------     298
CAMNT_0047551615_Pseudo-nitzschia_fraudulenta            MELLLK-------     241
CAMNT_0013109375_Pseudo-nitzschia_pungens_cingulata      LERLL--------     281
CAMNT_0013140303_Pseudo-nitzschia_pungens_pungens        LERLL--------     195
jgi_Fracy1|272356_Fragilariopsis_cylindrus               LERLL--------     97
CAMNT_0041255159_Fragilariopsis_kerguelensis_L2-C3       FERLL--------     284
```

Supplementary Figure 9. MRP3 protein sequence alignments. A. Alignment of the predicted proteins translated from the four *MRP3* alleles. **B.** Alignment of the predicted proteins translated from the *MRP3* homologues identified in public databases. * indicates positions which have a fully conserved residue, : indicates conservation between groups of strongly similar properties, . indicates conservation between groups of weakly similar properties.

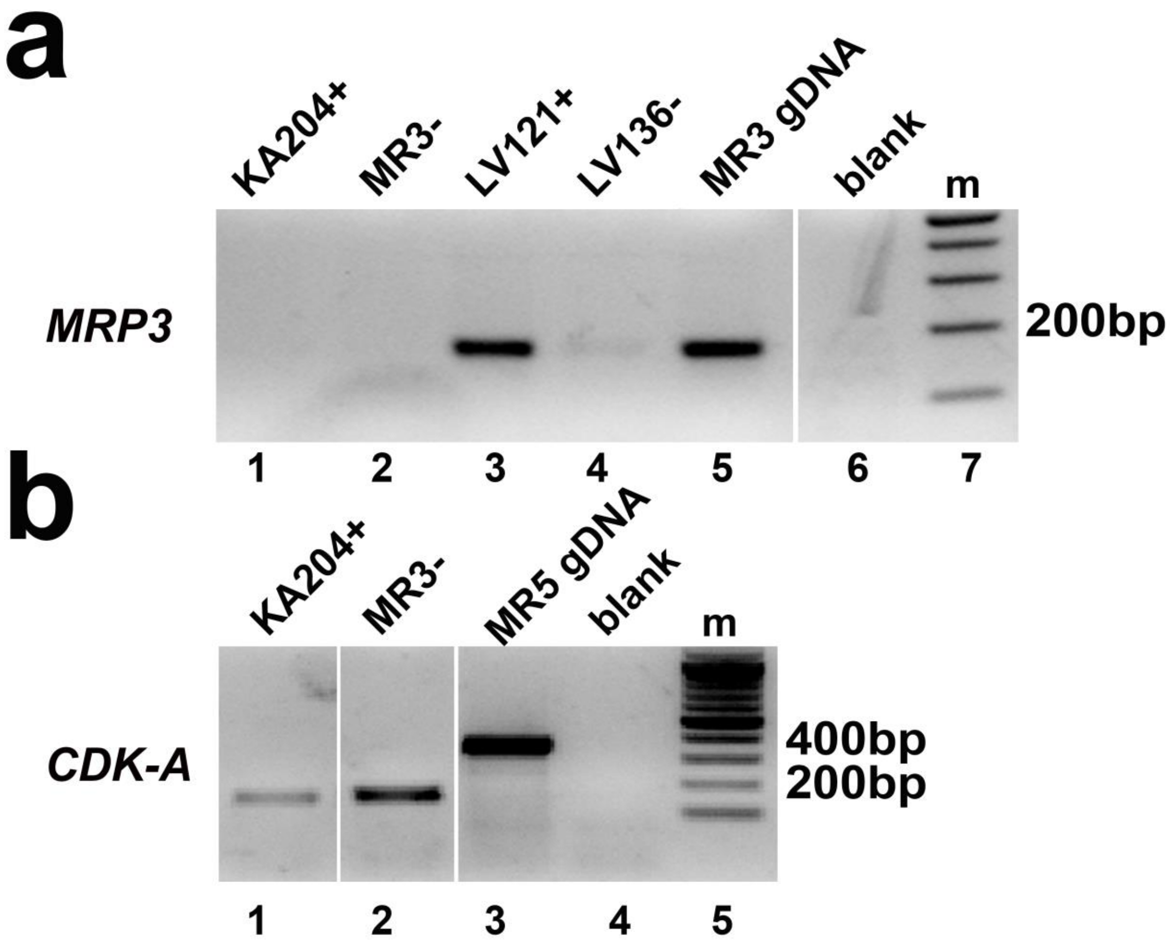

Supplementary Figure 10. ***MRP3*** **expression in strains >SST. A.** PCR amplification of an *MRP3* fragment from the cDNA of two strains >SST (lanes 1-2) and of two strains <SST (lanes 3-4). gDNA is used as a control for the reaction (lane 5). **B.** PCR amplification of an intron-containing fragment of the control gene *CDK-A* from the cDNA of the same strains >SST shown in a. + indicates MT+, - indicates MT-.

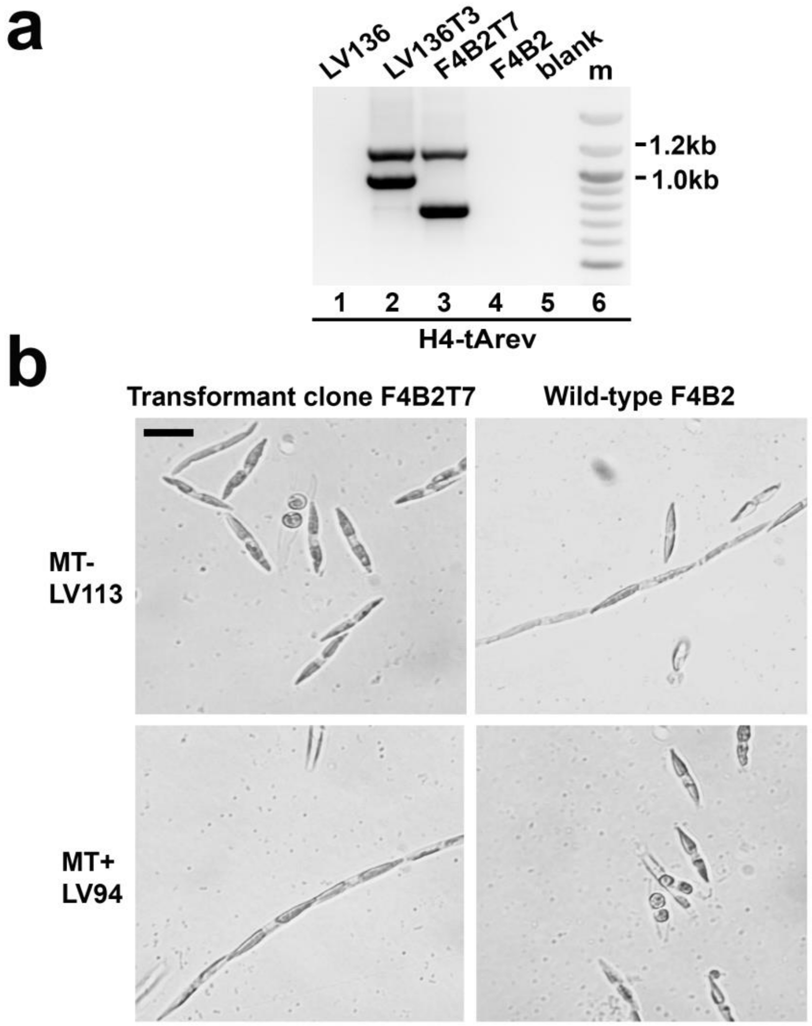

Supplementary Figure 11. **Sex reversal in the transgenic strain F4B2T7. A**. PCR showing integration of full length NAT resistance gene and of the *MRP3* transgene in the genomic DNA of the LV136T3 transformant (lane 2), and of the *Sh-ble* resistance gene and *MRP3* transgene in the genomic DNA of the F4B2T7 transformant (lane 3). Note that the plasmids used for transformation all have the same H4 promoter and the same FcpA terminator, amplification with a primer in the promoter and a primer in the terminator yields a 1.2 kb band for the *MRP3* transgene, a 1 kb band for the NAT transgene and a 0.8 kb band for the *Sh-ble* transgene. **B.** Images of crosses of transformant strain F4B2T7 and of wild-type MT- strain F4B2 with the MT- strain LV113 and the MT+ strain LV94. F4B2T7 cells mated with the MT- strain producing gametes, whereas they did not mate with the MT+ strain. The opposite occurred for the wild type strain F4B2. Scale bar= 20 μm.

a

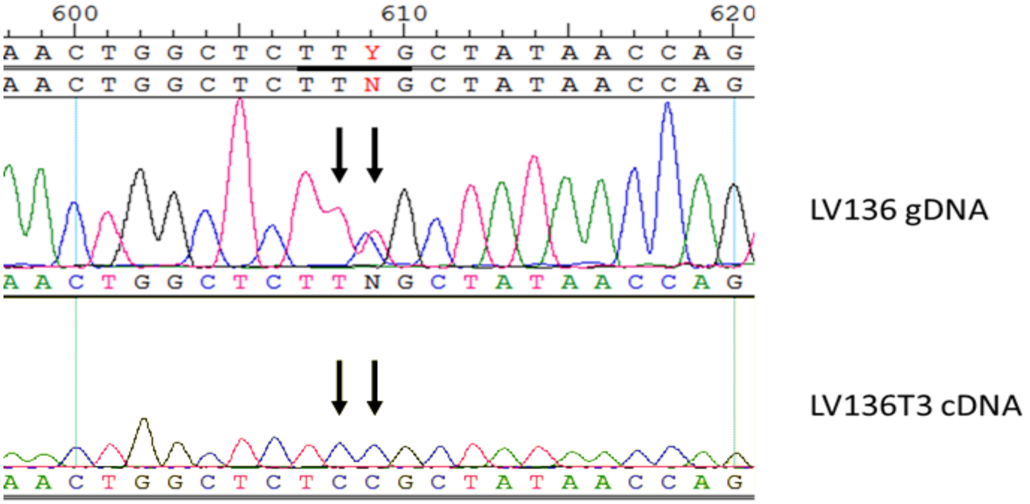
600
610
620
A A C T G G C T C T T Y G C T A T A A C C A G
A A C T G G C T C T T N G C T A T A A C C A G
A A C T G G C T C T T N G C T A T A A C C A G
LV136 gDNA
A A C T G G C T C T C C G C T A T A A C C A G
LV136T3 cDNA

b

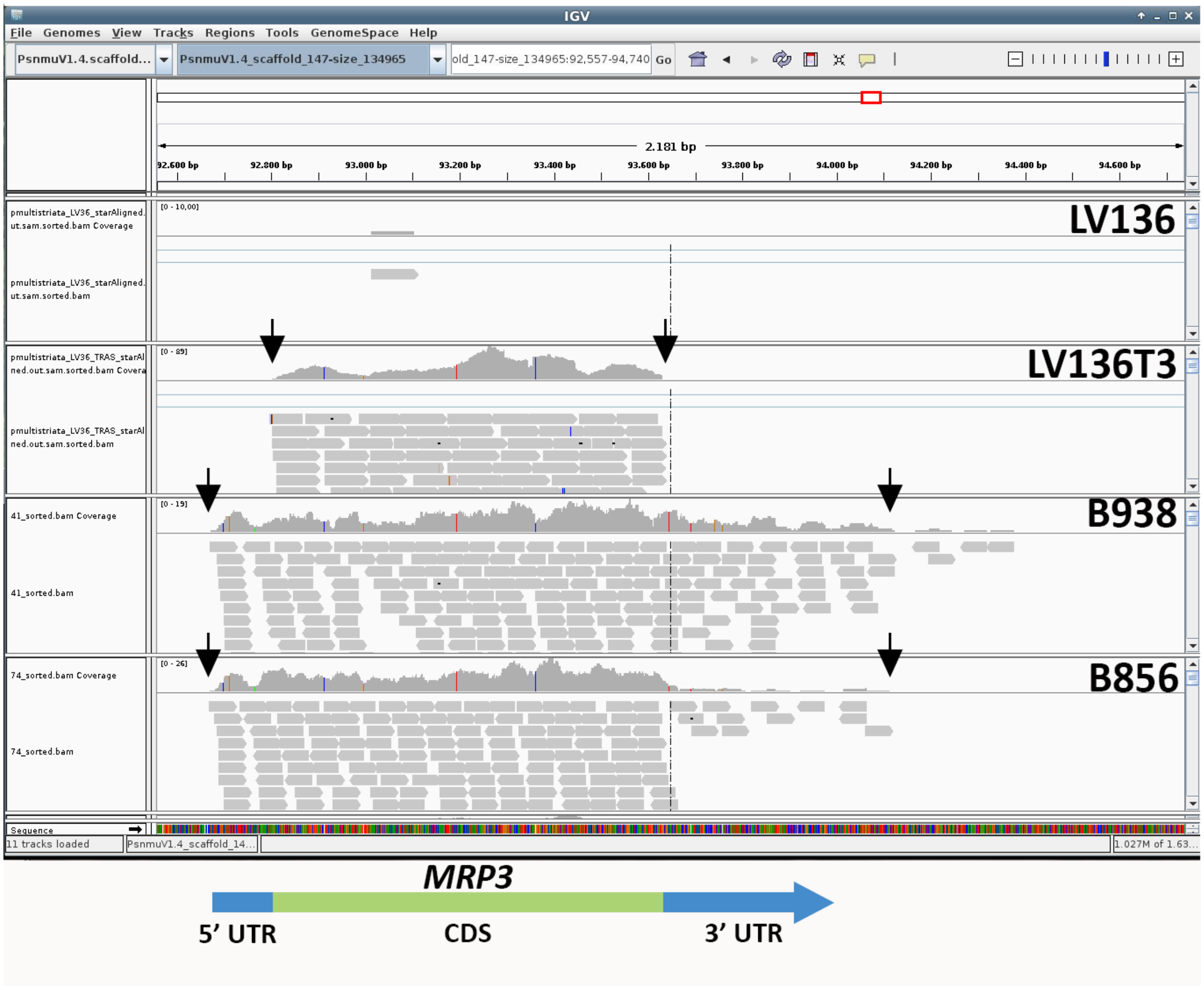
IGV
File Genomes View Tracks Regions Tools GenomeSpace Help
PsnmuV1.4_scaffold_147-size_134965
2.181 bp
LV136
LV136T3
B938
B856
41_sorted.bam Coverage
41_sorted.bam
74_sorted.bam Coverage
74_sorted.bam
Sequence
11 tracks loaded
MRP3
5' UTR
CDS
3' UTR

Supplementary Figure 12. Exogenous expression of *MRP3* in the transgenic strain LV136T3. A. The *MRP3* transcript expressed in the LV136T3 strain (bottom track) has the sequence of the transgene inserted and not of the endogenous gene (top track). The transgene was obtained by cloning the LV149 strain transcript (see Results). Note that LV149 and LV136 have different alleles with two consecutive SNPs (indicated by the two arrows). LV149 has the A/M genotype T**YC**G (see Figure 3B), while LV136 has the B/N genotype T**TY**G.
Also note that the sequenced fragment of LV136 gDNA has been amplified with forward primer F147p2 which is outside of the *MRP3* CDS. Therefore, it only amplifies the endogenous locus and not the transgene which is inserted randomly in the genome.
B. IGV visualization of the RNA-seq reads obtained from strains LV136, LV136T3, B938 and B856 mapped to the scaffold 147 region where the *MRP3* gene lies. Note that no reads are present for the MT- strain LV136 as the gene is not expressed, and that the reads present in the transgenic strain LV136T3 correspond to the CDS of the gene only, in contrast to the wild type MT+ strains B938 and B856 in which reads can be found also for the 5' and 3' UTRs. Vertical black arrows indicate the start and end of the transcript in each strain. Gray peaks indicate the number of reads (indicative of the levels of expression), colored vertical bars indicate nucleotides that differ with respect to the reference genome sequence, gray arrowheads are single reads mapped to the genome. A schematic representation of the transcript with its 5' UTR, CDS and 3' UTR in their approximate position in the genome is shown below.

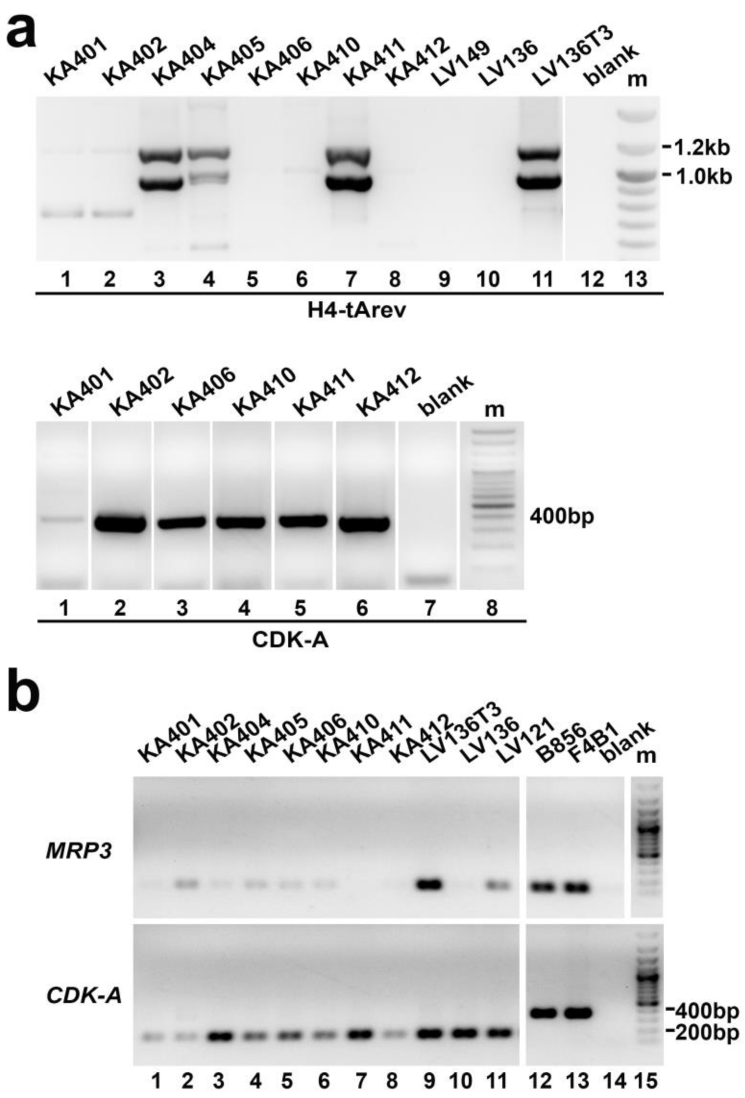

Supplementary Figure 13. ***MRP3*** **in the LV136T3 F1 progeny. A.** PCR screen to detect the presence of transgenes in the genomic DNA of the LV136T3 transformant F1 progeny. Amplification with a primer in the H4 promoter and a primer in the FcpA terminator yield a 1.2 kb band for the *MRP3* transgene and a 1 kb band for the NAT transgene. Note that the two plasmids used for transformation have the same promoter and the same terminator. In the top gel, lanes 1-8 contain F1 strains, lane 9-10 contain two wild type strains, lane 11 contains the LV136T3 transformant. As a quality control for the DNA, in the bottom gel the amplification of a control gene, *CDK-A*, is shown for the F1 strains that were negative for the presence of transgenes in the top gel. **B.** *MRP3* expression in the F1 progeny. Top gel, PCR amplification on the cDNAs obtained from the F1 progeny (lanes 1-8), from the LV136T3 transformant (lane 9), from the non-transformed LV136, and from an MT+ strain used as control. Lanes 12-13 contain gDNAs used as controls for the reaction. In the bottom gel, amplification of an intron-containing fragment of the control *CDK-A* gene is shown for the same samples.

>TRINITY_DN16811_c0_g1_i1
ATTCATCGATCACAAATATTCACGACAAAATCACTTTCAAATCAGAATTCATACAGATTC
ACAATAACGAAAATTGAAACATTCAAAAGGCCATTGAAAGCAAAATCGAGAGCCAAAAAC
ATATTGCAAACGATTTTCGTTTATTCATCATGTCACGATACAATCCAAAAGCCACCATCC
GTGAAGACGAAGAGTATCTCGATGCCGAGGTGGCTCCAAGAACTCCGGAAAATTCTGTAG
TGACGGCCGGAGTCGTCCTATCTTCGCCTTCTACGACCATTGGCCGCGAACAACATCCTG
ACCAAACCAATCAAGTCCTCGAATCCCAGTTTGCCAGCAAAGACTTTGATGACGACTTTA
ACAAGAAGAGAAACTTTAGTTTTGACTGGTTCATTTGGACCATGTTGGGAATGACCATTG
TGCTCTTGACCTTGGTTGGATATGGATTCGGTTCGGGCCTTTATCTTCCCAACAAGACAA
CTGTTGGCGGTGATGCTATTGCCAACTCCAACGATGCCGCAAGTCTTGCTGCGAGTGATG
GAAGCCTTTCCGAAGGAGAAACGCCCGCTCTTTCTCTAACCAACCCAGAAACCAATCGTC
AAGATTACAAGTACAGCATCATGACCCCCTTGGGCTTGCCCATTGTCATGGAAGGTGACT
CTGCCCAAGCTCAGGCGATTGATTGGCTGGCCTTTGACGACGAACCCCTCTTTGACCCAA
ATGCGATGGAAACCCAGGAAGACCAACACAAAGAGCGATTGACACAGCGCTATGCCTTGG
TGGTGTGGTATTTTGACCAGGGTGGCCCAGCCATGTGGACAACCTTGAATCGGGAAGAAT
CTGCCGGATGGATTGTGAACGGTGCCGGGGTTCACGAGTGCAACTGGAGGGGAATCGATT
GCGACTACAGCGAAATCAGTGATGGGAAAGTCATTGGACTTCGGCTATCGCCTATTGGCG
GGCTCTTGTTGACGGGTTCCTCCGTGTCTTCCGAGCTAGGGTTGCTGACAGGCCTCCAAC
GCATCGATTTTGCCGATCAGCGCATTCAGGGGAAGATTCCAAATTCATGGGCCCTCCTGA
CCAATCTTGAAACGGTGGTTCTTTCTGACAACAAGTTGCAATCGACGATTCCCGATTGGA
TAGGGGGATGGACGAACCTTCGGCATCTAGCGCTGGATGGAAATCAGTTGTACGGAACAA
TTCCCTCGTCCTTTGCCACTCTCCAAAAATTGGAAAGACTAGAGCTACAACAGAATCCCC
AGCTCCGGGGTCCATTTGAAGTATTGTTTTCCCACAACAATATGACTTCCGGCCCTCTAA
AGACCCTCGAGCATTTAGATCTTTCCAACACTGATCTGGAGGGTGAGCTCCCCAGTACCA
CTCTCCCATCCCTCAAGTTTCTGCGAATGTGGAATCTCAATGGATTGGTAGGGACAATCC
CAACACAAATCGGGAGCTGGTCCAACCTGGAATACTTGAGCCTAATGGAAAACCAAAACA
TCATGGGGAGCATCCCCACCGAGTTGGGCCTACTGACAAATCTCGAAGCCATCGAGATCC
TCGACAGCTTCCTCATGTCTGGAACTCTACCAACCGAGCTGGGCAATCTCAACCTCAAGA
GACTCAACGTTCGTTACATAAACCAAACTGGAATCCTTCCCGTTGAGTGGTCCAATATTA
GTAGCCTACATGTTTTGGATGTCAGCAACAACCATCTGGAAGGGACGATCCCCCCACAAT
ACTCTCAACTCAGCCAGCTCGAGTTCTTTTTCTTCCAGTACAACAAGTTGACTGGAGAAG
TTCCCGGCGGAGTTTGTTCCCTCGAAAATCGTCAGGAATTTCTCGTTGATTGTGATAAGG
GAGTTGGAAGAGGCAATATTACTTGTCTCTGCTGTTCGTGCATTTGATCAGCAATGCTTC
TACAAGAGTAAATGCGGGTTCAAACTATAATGATGGAAGAATTAATGATACTAATAGCAC
TAAATTTCATATTTCCTCCATCGTTTTAAAATAAAGATAGCAGATTTCTTGAAATCAAAT
CGTGGAAATATCAGTT
>TRINITY_DN7331_c0_g1_i1
CACTGCGAACACAACGTCTCATGAGCACGAAAACTGAAATGCAATTTGAAAAGTAATGTT
CTAGCTACAATCGAAACGGCATTATTTTCTTCGCTACGTGTGATTAGACTCAATTTAAAG
TATGTCAGACTGTTCGAATAGTGAATGGGATTTCTATTTCGGACGGGGTGGTGGCTCGAA
CCAGAAGCGACAAGTTTCAAAAGCCAATAGTTTGGTGCAAGATTTGGCTGTTCGTTATAG
TTACTGTAGACAAACTGAGAAAAGGCTCTTTGCAAAGAACGAAGTTTACGATACTGTTGT
TAACAATGGCGGCACTTTCTTTCTTGTTACCAATAAAGAATTCATTGACGTCACGAAGGA
CTTTGACGATACTATAAATAGGATCATGCAAAGCTTTCGAGACATCAATAAAATTTGCAA
AACAACATCCCAACCAGTATCAACAAGGGCTGAAGCGAAAAAAGTGCCGTCGCAGAAAAG
ATCATCTCCGATTCTAATGCCAACAGCTCGAGACGCGAACAGAAAAAACATGCAATCCAC
GAGTGTCAAAACCAAAAGAGTGTCATTAATCAAGAAATCACCTCGTCCAAAGCGTAGATG
TATACGGCCCAGTATAATCGAACCGCCTGTTATTGAATGCGTGAGGGATGCCCTCAGAGT
TGTCGACGCCGACAAAATAAAGCAAAATGGGAAAACACAGATCAGTGTCGGGGAGATACA
TACTACTTCATGCGATGATGGAACATCCAAGAGCAAGTTCGATGAAAGGGGTCTTTGTCA
ACTGGCAGCAGATGATGCATTTGAGAAAATGGTCGTGGAACCACCAAAGCTAGATAATTC
TTTCAGTCAATTGATCATGACGGATGAAATCAAAGCGCATCTTCCAACTGACTCACAACA
GTCGGTCAATCTCAATCTTCATTCAAGAGTACAACGTCTAGAGAACCTGGTAGCGATGCT
AATGCAACAAAAGAACGATGAAATGGAACTACTGTTAAAATAGGTTCTTTTTCTTTGCTA
ATCTGAGGAAGATTGTGTGTTGTGGCTGTGTTGATACTGGTGCTGGTGGTACAAGTAGCA
GTTTTGTTGCCATTCTTCAGTTTACGCTATGTCTATCGTTTAAATCGCGCCGCGTTTTTC
AAGACCCGGGCATGCATCATTCAGTCGACATATCATTTCTGAGGTATTTGAAACAGAGAC

```
ATGAGGATTTGGGTTAGTAAATGTCGTCTAACGGGCAGACAAATTTGGAGCGATGCTTAA
GGATTCTAATCACAATAGAGTACAATCGCATTTAGCAAAGATTTATATCATCGCAAAAAA
AAAAA
```

Supplementary Figure 14. *MRP2* and *MRP3* nucleotide sequences in *Pseudo-nitzschia arenysensis*.

Supplementary Note 1: Uncropped gel images

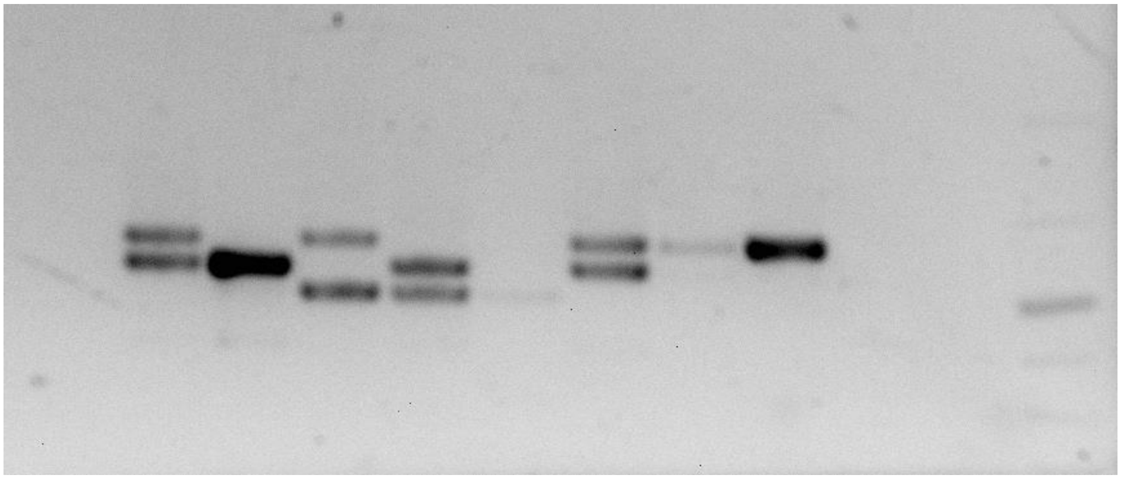

Uncropped gel Fig. 2b

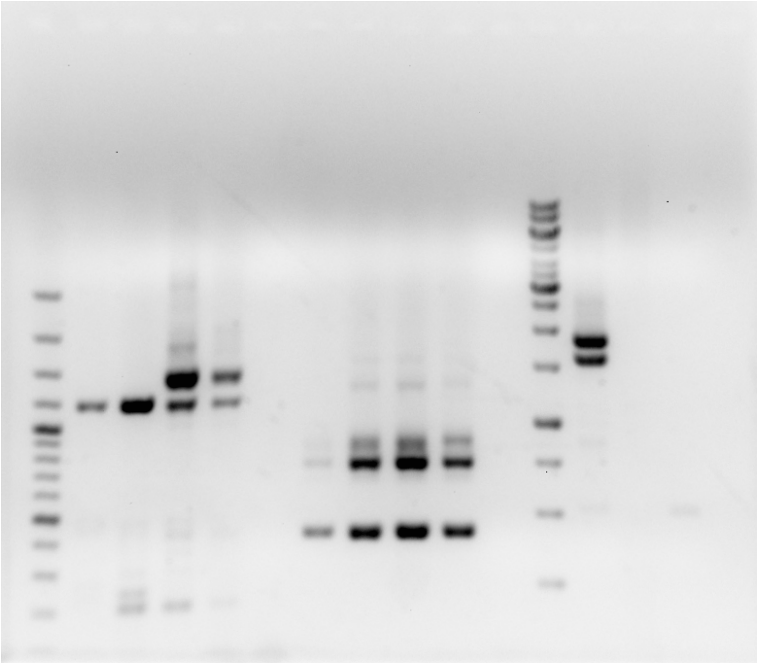

Uncropped gel Fig. 2c-d

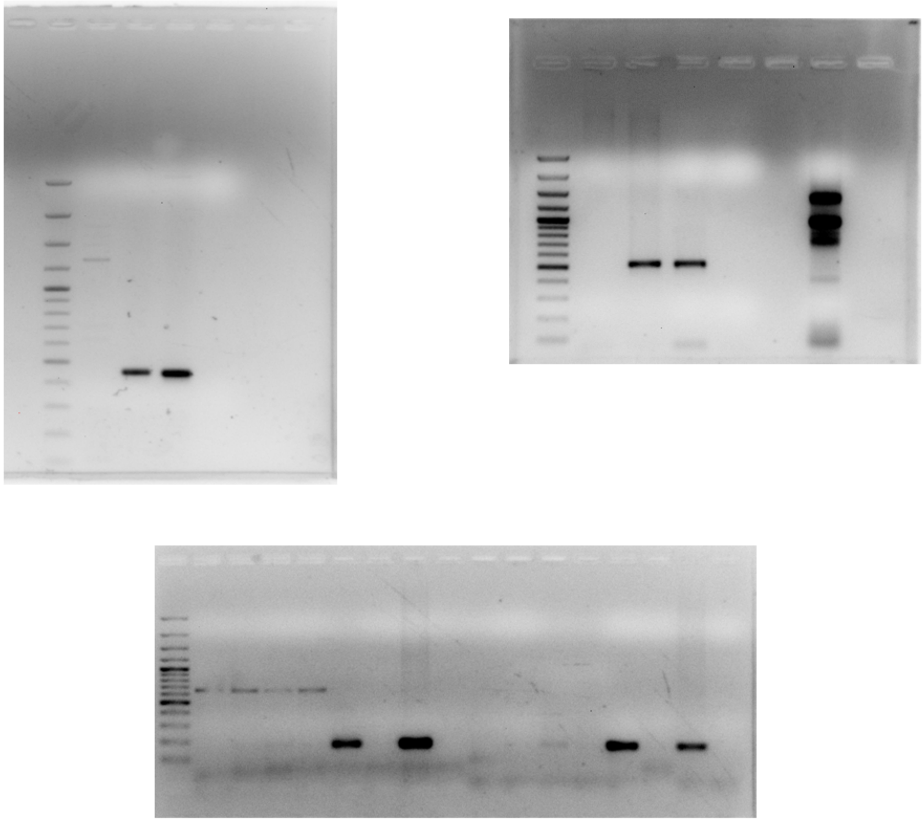

Uncropped gels Fig. 4a

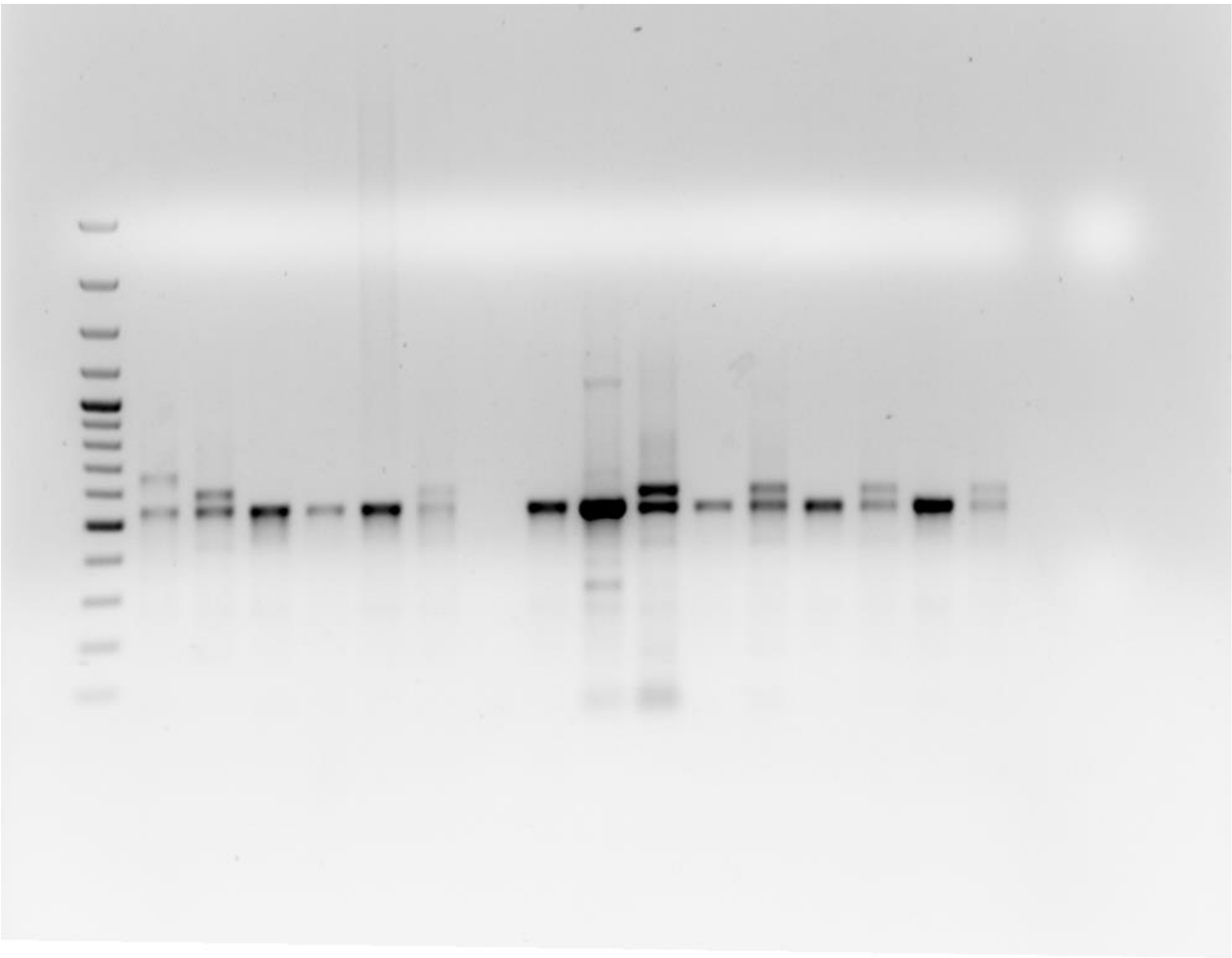

Uncropped gel Fig. 4c

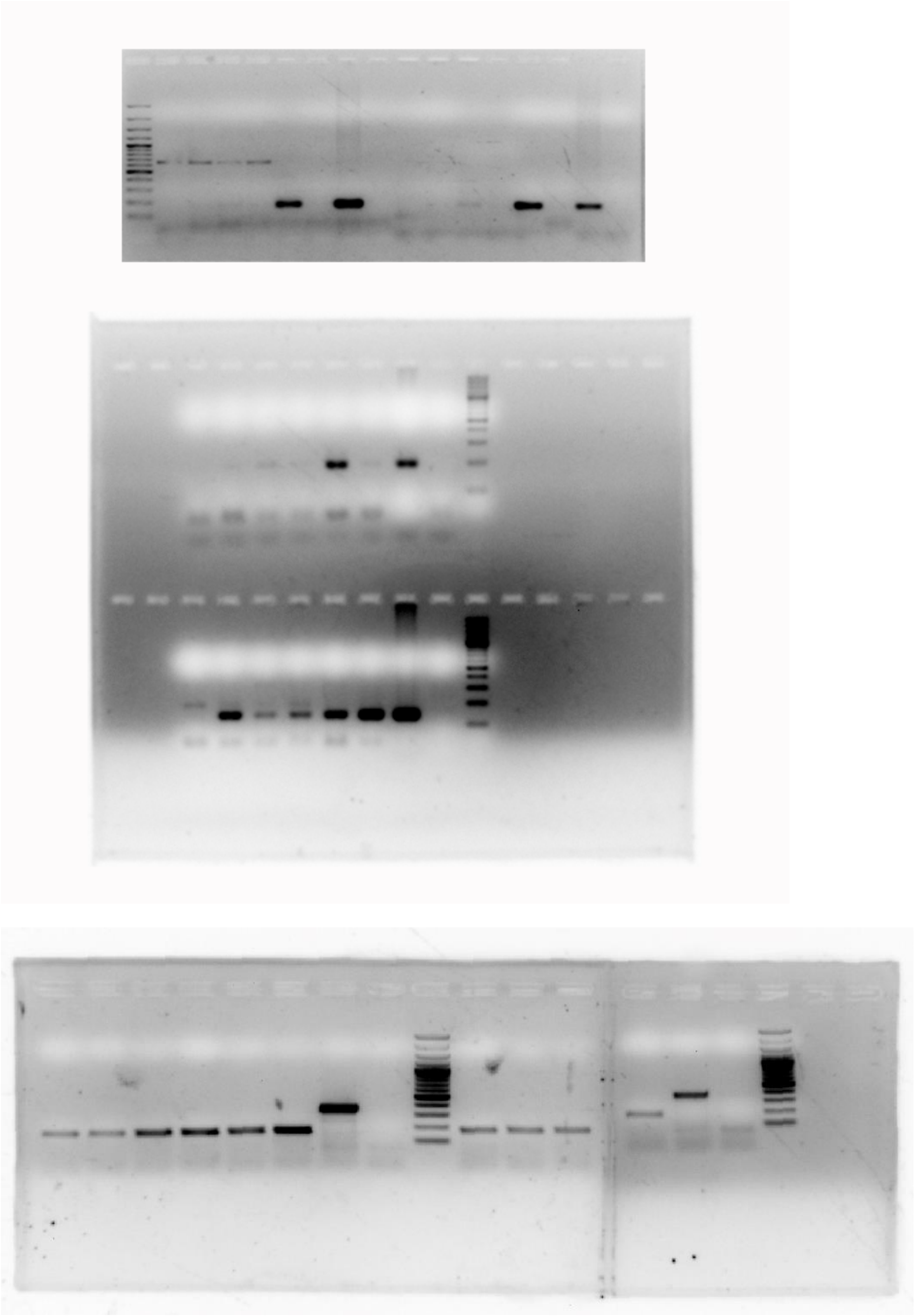

Uncropped gels Fig. 4d

Supplementary References

1. Basu, S. *et al.* Finding a partner in the ocean: molecular and evolutionary bases of the response to sexual cues in a planktonic diatom. *New Phytol.* **215**, 140–156 (2017).

2. Mock, T. *et al.* Evolutionary genomics of the cold-adapted diatom *Fragilariopsis cylindrus*. *Nature* **541**, 536–540 (2017).
