# Supplementary material for: MRP3 is a sex determining gene in the diatom Pseudo-nitzschia multistriata
Source: Nat Commun. 2018 Nov 28;9:5050. doi: 10.1038/s41467-018-07496-0 (PMC6261938; doi:10.1038/s41467-018-07496-0)
Supplement: Supplementary file 2 — Description of Additional Supplementary Files [file 41467_2018_7496_MOESM2_ESM.pdf]

Description of Additional Supplementary Files

File Name: Supplementary Data 1
Description: **TblastN output using the MRM1 protein as a query against the mmetsp_nucleotide database.**

qseqid Query Seq-id
sseqid Subject Seq-id
qstart Start of alignment in query
qend End of alignment in query
sstart Start of alignment in subject
send End of alignment in subject
qseq Aligned part of query sequence
sseq Aligned part of subject sequence
evalue Expect value
bitscore Bit score
score Raw score
length Alignment length
pident Percentage of identical matches
nident Number of identical matches
mismatch Number of mismatches
positive Number of positive-scoring matches
gapopen Number of gap openings
gaps Total number of gaps
ppos Percentage of positive-scoring matches
frames Query and subject frames separated by a '/'
btop Blast traceback operations (BTOP)
stitle Subject Title
qcovs Query Coverage Per Subject
qcovhsp Query Coverage Per HSP

File Name: Supplementary Data 2
Description: **TblastN output using the MRP2 protein as a query against the mmetsp_nucleotide database.** See legend to Supplementary Data 1.

File Name: Supplementary Data 3
Description: **TblastN output using the MRM2 protein as a query against the mmetsp_nucleotide database.** See legend to Supplementary Data 1.
